# Robust, scalable, and informative clustering for diverse biological networks

**DOI:** 10.1186/s13059-023-03062-0

**Published:** 2023-10-12

**Authors:** Chris Gaiteri, David R. Connell, Faraz A. Sultan, Artemis Iatrou, Bernard Ng, Boleslaw K. Szymanski, Ada Zhang, Shinya Tasaki

**Affiliations:** 1 Department of Psychiatry and Behavioral Sciences, SUNY Upstate Medical University, Syracuse, NY USA; 2 Rush Alzheimer’s Disease Center, Rush University Medical Center, Chicago, IL USA; 3 Department of Neurological Sciences, Rush University Medical Center, Chicago, IL USA; 4 Rush University Graduate College, Rush University Medical Center, Chicago, IL USA; 5 Department of Psychiatry, McLean Hospital, Harvard Medical School, Harvard University, Belmont, MA USA; 6 Department of Computer Science, Rensselaer Polytechnic Institute, Troy, NY USA; 7 Network Science and Technology Center, Rensselaer Polytechnic Institute, Troy, NY USA; 8 Academy of Social Sciences, Łódź, Poland

## Abstract

Clustering molecular data into informative groups is a primary step in extracting robust conclusions from big data. However, due to foundational issues in how they are defined and detected, such clusters are not always reliable, leading to unstable conclusions. We compare popular clustering algorithms across thousands of synthetic and real biological datasets, including a new consensus clustering algorithm—SpeakEasy2: Champagne. These tests identify trends in performance, show no single method is universally optimal, and allow us to examine factors behind variation in performance. Multiple metrics indicate SpeakEasy2 generally provides robust, scalable, and informative clusters for a range of applications.

**Supplementary Information:**

The online version contains supplementary material available at 10.1186/s13059-023-03062-0.

## Background

Clustering makes big data in biology tractable by defining groups of related objects that can be treated collectively. Clusters often correspond to specific biological functions, while reconfiguring clusters can induce adaptation or novel functions [1–6]. Due to these functional properties, and density of interactions within them, biological clusters are treated as robust building blocks that are expected to be replicated across related studies. Since the appeal of clusters stems in part from their apparent robustness, it may be surprising that clustering algorithms have several limitations that can skew a study’s conclusions. For instance, many clustering algorithms require the number of clusters to be supplied by the user, when this would ideally be learned from the data, in order to prevent circularity [7]. There may not even be a number of “true” clusters [8, 9], as many biological systems have multiscale organization [10–14]. Attempts to define “good” clusters **—** i.e., with many internal connections and few external connections [15] **—** have mathematical limitations [16–19] that can decrease reproducibility in biology. For instance, the same data set can be partitioned into clusters that both receive high modularity scores, but those solutions can be very different from each other [20]. There are attempts to address these flaws [21, 22]; however, even these efforts confront hard limits to defining a universally superior metric [23, 24]. Similarly, no clustering algorithm will be universally optimal across all networks [25], and no single metric will always be able to capture all aspects of realistic clusters [26]. These limitations on robustness can affect the interpretation of biological data, as the essential point of clustering is to provide high-confidence groups.

The litany of limitations to clusters stands in contrast to its ubiquitous application in biology. One avenue to address this situation is more diverse testing on varied types of networks [27], avoiding declarations of universal superiority based on a single data set or data type. A corollary is reducing the need for “tuning” parameters, needed to obtain “reasonable” results, which can lead to circularity. Therefore, we test several popular clustering methods in biology, and a proposed consensus clustering algorithm with minimal tuning, on a highly diverse set of networks, monitored by multiple quality metrics. While our algorithm is intended for application to common biological data types, we also test it on the Lancichinetti-Fortunato-Raddichi (LFR) benchmarks [28] to assess its generalizability. This is an uncommon test for algorithms intended for biology, though standard for methods originating in computer science. Furthermore, we vary every network-generation parameter in LFR (such as connectivity distributions) to provide a wide-ranging pool of networks, instead of potentially skewing results by using a specific set of networks. Because all network metrics have limitations, we assess the quality of partitions with multiple cluster quality measures. In addition to LFR, we test SE2 on multiple common biological data types. By monitoring the performance of several algorithms across bulk gene expression, single-cell, protein interaction networks, and even large-scale human networks, we can better estimate performance on novel data. Application to many data types requires us to address demands for special features that are somewhat specific to biology applications, namely, the ability to process negative edge weights and detect overlapping clusters. This broad assessment of clustering methods is unusual, essential to estimating the robustness when applied to future datasets.

### Features of SpeakEasy2: Champagne

**Fig. 1:**
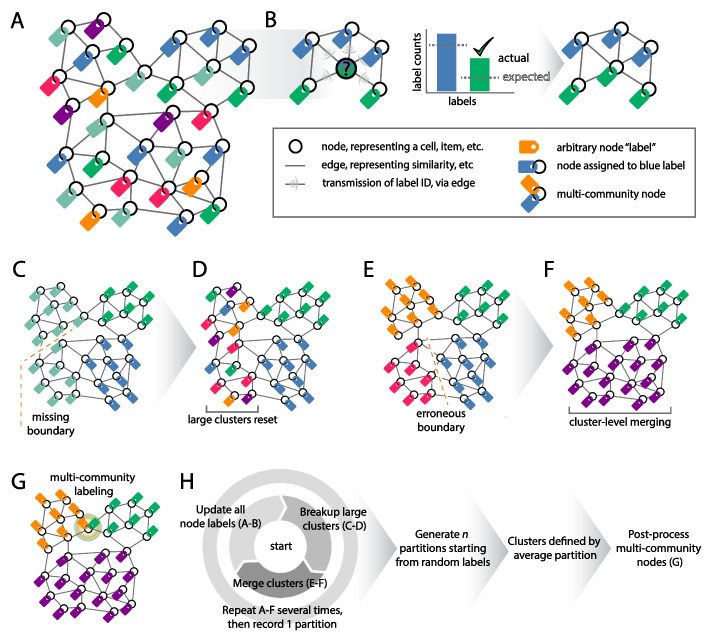
Overview of the clustering process in SpeakEasy 2: Champagne. **A** Each node in the network receives a random label, with the total number of labels less than the total number of nodes. **B** Each node updates its label to the most unexpectedly common label among its neighbors, accounting for the global frequency of each label. **C** Large clusters may mask multiple true communities. **D** Large ill-fitting clusters are split into random labels. **E** Stable sub-cluster configurations may occur which are not globally optimal. **F** By operating at the level of complete modules, suboptimal clusters can be split or merged to find globally optimal clustering states. **G** Multi-community nodes are identified based on those nodes which typically join a small number of distinct clusters, across multiple independent runs of SE2. **H** Overarching sequence of stages in SE2 algorithm, individually described in prior panels

The new method we propose is a “dynamic” approach to clustering that simulates communication between connected nodes, then derives a partition from this activity [29]. One type of dynamic clustering is called label propagation [30], in which limited labeled nodes propagate those labels to connected nodes. Originally, label propagation algorithms required some correctly labeled nodes. However, recent variations do not require any initial labels to be known [31]. The main feature separating the original SpeakEasy algorithm [32] from other label propagation algorithms [33] was a normalization term, which prevents large clusters from expanding, simply because they are large and non-specifically in contact with many nodes. Here, we develop a related algorithm, SpeakEasy 2: Champagne (SE2), which retains the core approach of popularity-corrected label propagation, but aims to efficiently reach a more accurate end state (Fig. 1). In particular, the changes increase accuracy by escaping from label configurations that become prematurely stuck in globally suboptimal states. To avoid this problem, SE2 utilizes a common approach in dynamical systems: making larger updates to jump out of suboptimal states, specifically using clusters-of-clusters, which allow it to reach configurations that would not be attained by only updating individual nodes. In contrast to most label propagation algorithms, which initialize with one unique label per node, SE2 increases runtime efficiency by initializing networks with far fewer labels than nodes (Fig. 1a), updates nodes to reflect the labels most specific to their neighbors (Fig. 1b), then divides the labels when their fit to the network drops below a certain level (Fig. 1cd). This reduced number of labels actually increases the opportunity for the label assignment to become stuck in suboptimal solution-states, but the more effective meta-clustering (Fig. 1ef) overcomes that possibility, as indicated in later results. Finally, nodes that have oscillated between labels over the course of many partitions are (optionally) identified as multi-community nodes (Fig. 1g). Overall, the operation of SE2 can be conceptualized as merging groups of nodes, balanced with a tendency for groups to burst open like bubbles when they have expanded too far (see “Methods”; Additional File 2).

### Alternative clustering approaches

We compare SE2 to several clustering methods selected for their popularity and representativeness of broader classes of clustering methods. There are several ways to group clustering methods [29], as methods can be defined by their historical field of application, their core mathematical operation, or by the metrics they attempt to optimize. As a comparison method within the “dynamic” category of clustering algorithms, we test Infomap [34]: a fast and popular algorithm that essentially utilizes a random walk through a network and defines clusters by compressing those walks into clusters of nodes that are frequently visited in sequence. Its operation also illustrates the blurred boundaries among classes of clustering algorithms, because it uses both a dynamic process (a random walker that traverses nodes) and optimal data compression (of the random walker’s path), so it could be considered an optimization approach — another major class of clustering methods.

In contrast to dynamic clustering algorithms, optimization approaches typically try to define a measure directly related to the quality of a final partition [15, 35], and then iteratively modify proto-clusters to maximize the quality metric. While this updating could be seen as a type of dynamics, optimization approaches are focused on increasing a global quantity (some measure of cluster quality) and nodes are typically lumped into varying sets without any communication process among them. We test the related Louvain and Leiden algorithms as an example of an optimization approach, which in biology is commonly applied to single-cell RNAseq data. Typical weaknesses of these measures include the enormous combinatorial search space of all partitions and limitations of the quality metric they are trying to optimize [15]. For instance, even when an algorithm produces high modularity scores for a given network, there can be numerous other partitions with equally good scores that differ significantly from each other [20]. Thus, even excellent optimization algorithms can be short-circuited (in terms of reproducibility) by the choice of metric.

Instead of optimizing a metric that is basically an external description of a good partition, “model-based” approaches to clustering often start with a principled view of what constitutes members of a cluster. Where do these principles come from and how are they used to define partitions? In some cases, they are simply reasonable mathematical stipulations on how clusters should be compactly represented, such as in non-negative matrix factorization (NMF) [36], which we test. Some other model-based approaches contain generative models, capable of producing synthetic networks. In these cases, cluster detection in real networks is focused on extracting the types of clusters produced by the model, such as the stochastic block model (SBM) [37]. Thus, for model-based clustering methods, the accuracy of their results is linked to how well their model of clusters actually conforms to the real data at hand. Once again, this particular class of algorithms is not entirely unique, as certain block models are equivalent to global optimization of modularity [38].

Machine learning (ML) clustering methods stand in contrast to generative models and their focus on the theoretical origins of clusters. As with many ML applications, it may be possible to achieve good results while entirely ignoring how a particular network was generated. Most approaches seek clusters via a compressed representation of an input network that is ultimately useful for clustering. These representations are typically generated via the hidden layers of an autoencoder [39, 40], or by graph embedding mechanisms [41–44]. In both cases, the focus of these methods is to compactly represent the major aspects of each node, which can be its expected neighbors or any other properties [45]. After doing so, nodes which are nearby each other in the compressed space will be nominated as clusters with relatively primitive methods, such as k-means or SVM. Such approaches can process LFR networks [39, 46] and are generally able to incorporate additional node properties (beyond connectivity) that are not considered by traditional graph-based community detection methods. However, deep learning methods face the usual challenges of determining cluster number, and signed edges, while general lack of scalability and raw performance [47] limit their use in biological community detection applications as compared to traditional community detection on graphs.

A final category of clustering methods could be an omnibus of methods with unusual capabilities, aimed at biological with specific origins or features. Unusual capabilities would include (1) the ability to bicluster samples and variables simultaneously, (2) overlapping clustering that place objects into multiple communities [48], or (3) time-series methods that utilize a sequence of networks to extract meaning from edge dynamics [49–51]. Methods aimed at a particular type of biological data include methods for clustering gene expression [52]—which we test here—or cell differentiation data [53]. Many clustering methods that are popular in biology fall into this category, as they are heuristically employed on the basis of providing useful results on a specific setting, such as WGCNA in the context of gene expression data. However, their performance on new data types is unclear since they are rarely tested on synthetic datasets, such as LFR.

## Results

To robustly assess the accuracy of several clustering algorithms, we apply them to LFR benchmark networks and several biological data types. The rationale for testing a clustering algorithm aimed at biological data on LFR networks is that the ground truth partition of these networks is known, whereas it is undefined in many biological data sets. Furthermore, features of LFR (long-tail degree distributions, etc.) are similar to some biological networks. We additionally test the method on large-scale networks and diverse classes of networks, some of which are arguably non-biological in order to anticipate results when applied to novel biology data types, which might have new features and tend to contain many nodes. Because clusters are multi-faceted entities, we deploy multiple metrics of success in each application to ensure that performance is related to the underlying data and not the particular metric. In contrast, most clustering algorithms are proposed based on performance in a small set of networks, despite theoretical and applied results indicating that a single clustering algorithm is optimal for all clustering applications [20]. Therefore, these extensive tests will describe performance on specific data types and describe which algorithms, if any, succeed broadly and are more likely to be useful on new data types.

### Disjoint cluster detection in synthetic networks

A straightforward measure of clustering performance is to compare inferred clusters to a network’s true clusters. Since true cluster membership in real-world networks is rarely available, the most common way to generate networks with known clusters. In the LFR approach, networks are initialized from perfectly isolated (known) clusters that are cross-linked to a varying extent. Algorithms then attempt to recover the true clusters from the noisy network. Several network properties, such as degree distributions and weight distributions, can also be controlled to emulate the properties of real-world biological networks [54]. These parameters are typically set to a single level for all tests. In contrast, to provide more generalizable results, we generate many diverse synthetic test networks by varying all parameters in the range of what is typically found in the literature.

There are two typical approaches to varying the amount of cluster cross-linking in LFR networks: in the first, the total weight of cross-linking edges and the number of cross-linking edges are co-varied; in the less common second approach, only the number of edges (not their weight) between communities is varied. For robustness, we test both scenarios, and for completeness, we also test networks in which the portion of cross-linking edges is constant but their weight varies. In each case, the networks tested contain all other combinations of all other parameters, providing a highly diverse set of 576 LFR networks with different topological properties, but always containing disjoint (non-overlapping) clusters. Using these networks, we compare the performance of our new method to five other clustering algorithms, each of which represents a distinctive approach to community detection, as described in the introduction.

There are several options for measuring the accuracy of cluster recovery relative to the true partition [22, 55]. The two most commonly deployed measures of accuracy in this setting, where cross-linking between true clusters is varied, are the adjusted Rand index (ARI—related to the size of cluster–cluster overlap between two partitions) and normalized mutual information (NMI—related to how much information one partition provides about another partition). We also deploy inferred measures of accuracy (such as modularity) that do not rely on comparisons to the true partition, but which are related to the number of within-cluster connections versus between-cluster connections.

**Fig. 2:**
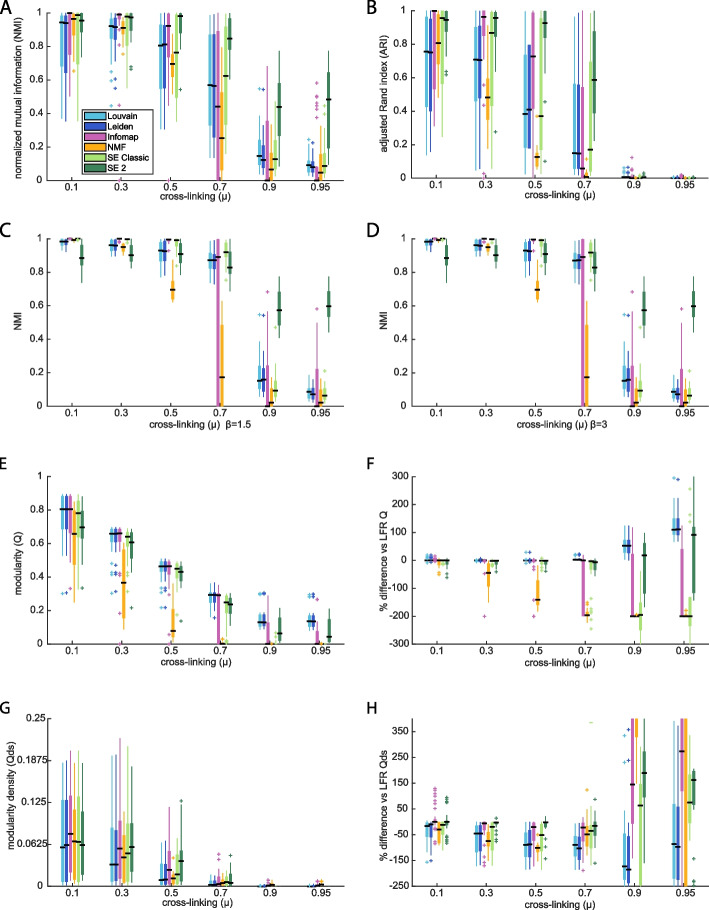
Recovering disjoint ground-truth clusters from LFR benchmark networks. **A** Recovery of original LFR communities, by several clustering methods, quantified by normalized mutual information (NMI), grouped by levels of cluster cross-linking (“μ”, *x*-axis). Cross-linking edge weights are co-varied with frequency of cross-linking for all results in this panel. **B** Recovery of original LFR communities, quantified by adjusted rand index (ARI). **C** Recovery of original LFR communities, by several clustering methods, quantified by normalized mutual information, for all networks with the weight distribution exponent (*β*) level equal to 1.5. **D** Recovery of original LFR communities, by several clustering methods, quantified by normalized mutual information, for all networks with *β* level equal 3. **E** Classic Newman’s modularity of inferred clusters. **F** Comparison of modularity of inferred clusters relative to the modularity of the ground truth solution. Modularity of inferred solutions may be greater than that of the true clusters (percent difference greater than zero) in noisy (high *μ*) networks. **G** Modularity density (“Qds”) of inferred clusters. **I** Comparison of modularity density of inferred clusters to that of the ground truth solution

The most influential variable within LFR networks is the fraction of cross-linking edges (“μ”), since this is a direct influence on the difficulty of recovering the original clusters. As in prior research, we co-vary the total weight of cross-linking edges along with their frequency, enabling us to create increasingly difficult cluster recovery challenges, with escalating occurrence and weight of cross-linking edges. As the number and weight of cross-linking edges increases, recovery of ground truth clusters decreases for all algorithms (Fig. 2a). Indeed, past *μ* = 0.5, there are more edges between clusters than within them, and at *μ* > 0.9, the original clusters can be qualitatively considered to be highly degraded. While both NMI and ARI show the expected trends with increasing cross-linking, results using NMI are less binary in the context of LFR, so we focus on those.

When cluster recovery becomes more challenging at levels of *μ* ≥ 0.7, we find that NMI scores of SE2 are significantly (*p* < e − 17) higher than the next-strongest method, Louvain (Fig. 2a), as are ARI scores (*p* < e − 11) (Fig. 2b). This indicates greater levels of cluster recovery across a range of network structures, which cover all combinations of all parameters in LFR, indicating that the performance advantage of SE2 by these metrics is not specific to a narrowly defined network topology. The increased performance over Louvain also indicates that maximizing modularity (Q) is not necessarily equivalent to optimal recovery of realistic clusters, as defined in LFR. The performance difference between SE2 and Louvain is most sensitive to the weight distribution parameter (termed “β” in the LFR code). Generating results at a single β-value can therefore have an outsized influence on perceived performance (*β* = 1.5 in Fig. 2c vs *β* = 3 in Fig. 2d).

Typically, cluster recovery in LFR networks is measured relative to the true clusters, but this perspective may be less relevant when the true clusters have been nearly destroyed by heavy cross-linking, in which case recovering them could actually be a flaw. Therefore, to provide an alternate assessment of algorithm performance in the case of high *μ* (where clusters are highly cross-linked and original configuration is degraded), we utilize modularity (Q) and modularity density (Qds). The latter measure corrects some mathematical flaws in modularity and can be thought of broadly as favoring more dense clusters. The modularity associated with the inferred communities falls as clusters become more cross-linked (Fig. 2e), due to the action of penalties for cross-linking in that measure, and modularity density behaves similarly (Fig. 2g). The modularity associated with the ground-truth partition might be expected to be a ceiling on performance; however, in the case of severely degraded clusters, community detection methods may be able to find communities superior to the true ones, amid the noise of rewired networks [56, 57]. Indeed, this is consistently the case at high *μ*-values, for Louvain and SE2, both in terms of modularity (Fig. 2f) and modularity density (Fig. 2h). The very highest relative modularity density measures are prone to instability as the raw values approach zero, which makes the raw values more reliable for that case (see Supplement). These tests indicate that an algorithm’s performance can vary depending on the perspective. For instance, SE2 shows significantly higher performance by NMI and ARI, Louvain shows significantly higher performance in modularity, and the methods are indistinguishable in terms of modularity density. The influence of network parameterization (controlling the properties of test networks) on perceived performance can be easily observed (Fig. 2cd), indicating the value of more diverse test sets.

### Overlapping cluster detection in synthetic networks

**Fig. 3:**
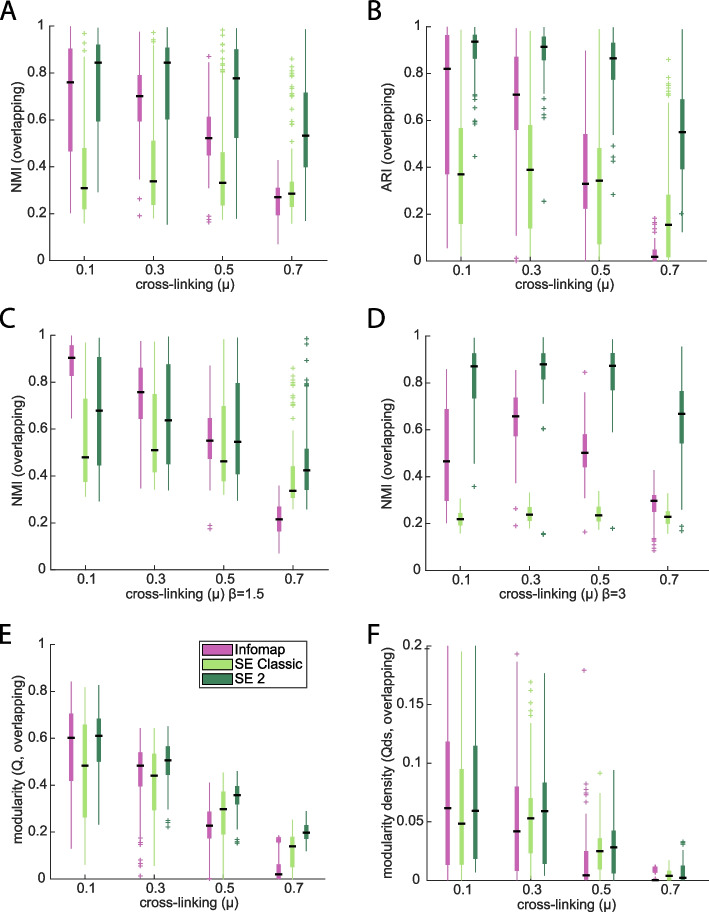
Recovering overlapping clusters and in LFR benchmark networks. **A** Recovering of true overlapping clusters in LFR benchmark networks, quantified by normalized mutual information (NMI). **B** Recovering of true clusters in LFR benchmark networks, quantified by normalized mutual information (NMI). **C** Correspondence of inferred clusters with ground-truth clusters, for a collection of LFR networks at various levels of cross-linking (*μ*), where cross-linking weight is scaled with *μ*, and weight distribution exponents (*β*) of all networks is 1.5. **D** Correspondence of inferred clusters with ground-truth clusters (same as panel **A**) but with weight distribution exponents equal to 3. **E** Classic Newman’s modularity for inferred clusters on LFR networks, grouped by proportion of cross-linking edge (μ). **F** Modularity density for inferred clusters on LFR networks, grouped by proportion of cross-linking edges (μ)

The synthetic test networks for overlapping cluster membership include additional parameters for the percentage of multi-community nodes (1, 5, or 10%) and the number of simultaneous community membership (2, 3 or 4). By varying these parameters, along with others used in the non-overlapping network test set, we generated 1728 LFR test networks with overlapping cluster membership. Because most clustering methods do not produce overlapping clusters (partitions with multi-community nodes), we compare SE2 to SE and Infomap. The ability to recover original clusters was substantially higher in SE2 vs. SE or Infomap across all levels of *μ*, using either NMI (*p* < e − 29, Fig. 3a) or ARI (*p* < e − 83, Fig. 3b) to measure cluster recovery. (LFR code was unable to output overlapping clusters for some parameters at *μ* ≥ 0.9.) Unlike the disjoint network results, these differences can be observed across the range of levels of cross-linking. These results are also broadly consistent with other frameworks for varying the amount of cross-linking between clusters—either with variable edge weights and constant number of cross-linking edges (*μ*) (Fig. S4), or with edge weights between clusters held constant (Additional File 1: Fig. S5), see Supplement for details on these tests. Absolute and relative performance of SE2 was most sensitive (subsequent to μ) to the exponent for weight distribution “*β*” (*p* < e − 22). While SE2 has significantly higher modularity (*p* < e − 36, Fig. 3e) and modularity density (*p* < e − 23, Fig. 3f) compared to SE, effect sizes are much smaller (~ 18 and 12%) than those observed in terms of NMI and ARI (50% +).

**Fig. 4:**
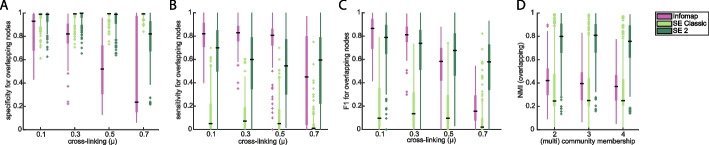
Identification of multi-community nodes in LFR benchmark networks. **A** Specificity of inferred multi-community nodes for true (LFR) multi-community nodes. **B** Sensitivity of inferred multi-community nodes. **C** Combination of sensitivity and specificity (F1) for identification of multi-community nodes, for varied numbers of split community memberships. **D** Correspondence of inferred clusters with ground-truth clusters (NMI, *y*-axis), for a collection of LFR networks wherein some nodes are assigned to multiple communities (*x*-axis)

Since SE2 is typically the most accurate method on disjoint LFR networks (those with non-overlapping clusters, Fig. 2), it might be unsurprising for SE2 to be the highest performer in the context of networks with overlapping clusters, as only fraction of nodes are multi-community. To specifically focus on the overlapping aspects of communities, we consider the sensitivity and specificity of the nodes each method nominates as multi-community, compared to the LFR ground truth. The original SE provided the highest specificity (Fig. 4a), but also the lowest sensitivity (Fig. 4b), while SE2 showed significantly higher (*p* < e − 94) joint sensitivity and specificity than other methods (Fig. 4c). Performance on overall cluster recovery (not just overlapping nodes) might be expected to decrease as nodes become split between an increasing number of communities, but SE2 shows sustained ability to detect nodes in up to 4 communities (Fig. 4d) at twice the rate of competing methods.

### Finding coexpressed gene sets in bulk gene expression

The central goal of clustering methods for gene coexpression analysis is to partition expression data into clusters of genes—typically referred to as “modules” in this context—with diverse function and phenotypical relevance. Such applications of clustering are a common and essential component of many systems biology and big data approaches to transcriptome data [58], in large part because they are tissue- and disease-specific and do not rely on existing ontologies of gene functions. While modules are defined without any reference to traits of the individuals or samples that provide the gene expression input, some modules may be found to be associated with these traits, which forms a starting point for understanding their collective function, causal structure, and disease mechanisms. All genes within trait-associated modules are further processed with computational and experimental methods to verify their function and the roles of specific genes. Modules can also represent other types of variables: for instance, the proportion of different cell types varies across samples, so genes that are cell-type specific may also induce coexpression modules [59, 60].

Several challenges exist to determining the best methods for identifying clusters of coexpressed genes (modules), because the usual tools—synthetic data generators, external validation, and abstract quality metrics—are very limited. The biological processes that lead to module generation are diverse [58] and no synthetic gene expression simulators produce modular expression patterns. Most coexpression clustering methods have been developed specifically for this data type and cannot be tested on LFR, as they require manual parameter tuning. It is possible to compute functional enrichment scores for each cluster, but the hierarchical nature of Gene ontology GO makes computing an overall score for the partition problematic. The annotation itself is highly skewed [61, 62], meaning that even true (novel) clusters might be scored poorly. Finally, abstract metrics such as modularity have never been shown to produce ideal functional enrichment. In this situation, where traditional methods of clustering method evaluation are less applicable, we assemble four different approaches to quantify how well SE2 detects coexpression modules.

We compare SE2 performance to WGCNA (weighted gene coexpression network analysis) [52, 63], which is the most popular gene coexpression clustering algorithm. This method is based on hierarchical clustering and assumes the input network will be scale-free. It was developed specifically to group genes measured in “bulk” (tissue-level samples are often comprised of mixed cells types) gene expression, as opposed to single-cell RNAseq. It is *not* possible to test WGCNA on LFR benchmarks due to multiple manual parameter settings and reliance on a particular connectivity distribution. Therefore, we examine WGCNA performance vs. SE2 in several tests using real gene expression data: (1) the stability of results across different sample sizes (which is crucial, as many biological sample sizes have hard limits due to sample availability), (2) the relationship of modules to key phenotypes, (3) the abstract quality of their output, and (4) the magnitude and diversity of biological enrichment in the modules produced by each method.

The sample size at which stable clusters may be obtained is an important component of clustering gene expression because many collections of tissues require years to assemble and cannot be easily augmented. Thus, understanding how few samples are required for stable clusters to emerge is a common cost/benefit question. To evaluate the stability of clustering output in small sample sizes, we output clusters from WGCNA and SE2 for datasets with varied sample sizes, spanning a range of the smallest to largest transcriptome data sample sizes. The input for this operation is gene expression measured in the dorsolateral prefrontal cortex from the ROS and MAP cohorts [64], currently the largest bulk gene expression dataset (*n* = 1207). This large sample size alleviates the propensity for larger subsamples to have greater overlap, and we always compare clusters from sampled sets to a held-out set of 400 samples.

**Fig. 5:**
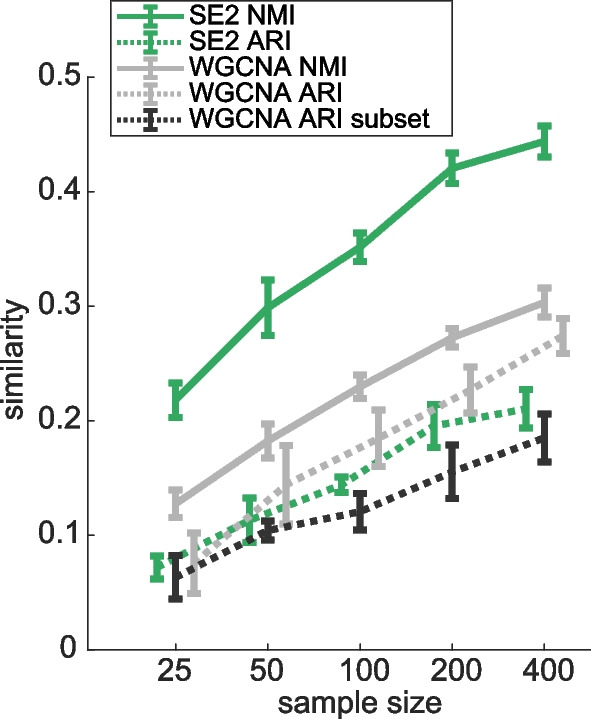
Stability of bulk RNAseq modules (gene expression clusters) derived from varied sample sizes, quantified by comparison to held-out data

We examine the stability of partitions between 400 held-out samples and partitions generated from smaller groups of samples (generated from random draws of samples, of a given size). For both methods, as sample size increases, the similarity of resulting partitions with the data partition increases (Fig. 5). Median NMI between partitions from small subsets of data and the partition from the held-out data is significantly higher (44% difference, across all levels of subsampling and replicates) for SE2 vs. WGCNA (*p* < 10^ − 9), while ARI was significantly (20%, *p* < e − 6) lower than WGCNA (Fig. 5). Differences in NMI and ARI are due to the large number of unclassified nodes from WGCNA (see Supplement).

To test the abstract quality of clusters by each method, we measure modularity “Q” [15] and modularity density “Qds” for all sample sizes [22]. The latter measure addresses two well-known limitations to classic modularity—the “resolution limit” in which modularity increases while counterintuitively merging small clusters with large clusters, and another flaw in which higher Q values can result from splitting densely connected clusters. Because we apply these tests to gene–gene covariance matrices, which include negative values, we adopt an extension to modularity that incorporates negative values/edges [65]. We find that median Q of the SE2 partition of the gene–gene covariance matrix is 268% higher than WGCNA (0.066 vs 0.023, *p* < 0.005) and Qds of SE2 is 200% higher (0.021 vs 0.011 *p* < 0.005). We find similar results in the next two largest brain-based RNAseq datasets (*n* = 259 temporal cortex from the Mayo brain bank and *n* = 318 frontal cortex from the Mt. Sinai brain bank, see Supplement).

To examine the practical influence of different coexpression methods on biological conclusions, we examine the sensitivity and stability of module-trait associations under various samples sizes. Specifically, we examine the magnitude of module correlations with cognitive decline as a function of sample size. We select this trait because it has the strongest univariate gene associations of any trait in the cohort. We vary the input sample size because a common concern in biology is if the sample size is sufficient for network analysis, and because there are often hard limits on sample sizes in biology. Both methods detected significant associations between cognition and the average expression of genes in a module (mean association of module with highest correlation, across all replicates for SE2: *p* < e − 11, WGCNA: *p* < e − 8) although the association was higher for SE2 (*p* < 0.05). Because an ideal method would nominate a consistent set of genes within the cluster most correlated with a trait of interest, regardless of input sample size, we consider the stability of genes in the module most correlated with cognition, as a function of sample size. In practical terms, stable module-trait associations mean that, as biological samples accumulate over a span of years, the same module will be consistently nominated in publications, as opposed to flip-flopping between various modules. To monitor for this stability, we compare the module that is most correlated with cognition in the held-out data to the module that is most correlated with cognition in disjoint subsets of samples. SE2 has a significantly greater proportion (*p* < 0.005) of instances where the gene members of the top cognition-associated module overlapped highly (*p* < e − 16) the gene members of the top cognition-associated module from the 400 held-out samples. This indicates the actual gene IDs of modules most-associated with cognition are more stable in SE2, even at small sample sizes, and thus have greater potential to be correct. Relatedly, we find the variation in the size of the top cognition-associated module (across subsets) is smaller in SE2 (p < 0.001), which also falls in line with the selection of a more consistent gene set associated with a trait of interest. The practical implication of this stable output for ongoing studies is that SE2 will more quickly settle and stay on the most disease-associated molecular system. We also repeated these tests using two other major phenotypes in Alzheimer’s—amyloid and tau levels—and found parallel results. While these results from real module-trait associations may not generalize to all datasets and all traits, they are a reasonable estimate given these datasets are the largest available, and results from coexpression clusters in this data have successfully driven experimental systems [60].

Genes may be influenced by multiple molecular systems [58] and have patterns of expression that are indeed correlated with multiple sources. Therefore, it could be helpful to assign such genes to more than one module. The nature of hierarchical clustering and WGCNA entails that genes are placed in only one module. We find that rerunning SE2 while allowing genes to be members of two modules results in 1548 genes (9%) labeled as multi-community nodes with no significant drop in the average significance of gene ontology enrichment as compared to disjoint output. The option to enable overlapping output is user-selectable.

### Finding cell types in single-cell gene expression data

In contrast to bulk tissue data, where the goal of clustering is to group genes, in single-cell data the goal of clustering is to group cells. First, cells are represented in a reduced data space to emphasize distinctions among cells. The data-reduced single cells are then converted into a network by linking each cell to some number of similar cells, according to some distance metric. This conversion to a sparse matrix is practically necessary, as few methods can process a full matrix between hundreds of thousands of cells. Clusters identified in this network are nominated as “cell types”. Downstream analysis typically associates phenotypes of interest with the number of these cell types, or expression within a cell type.

We evaluate single-cell clustering via hundreds of versions of the most well-studied datasets [66–70], each stemming from a different combination of preprocessing methods, in order to provide robust conclusions [71]. We evaluate the quality of clustering in two ways: in terms of graph community quality metrics (i.e., high modularity or modularity density) and by comparison to “gold-standard” cell types. Contrary to their name, the gold-standard cell types may not be independent definitions, as they are sometimes generated by clustering (see Supplement). Therefore, recovering the gold standards may be a marker of good performance, or just similarity to the original clustering method.

We cluster single-cell data sets, comparing SE2 to Louvain, which is the core of the popular Seurat method [72] (Seurat is essentially Louvain atop typical single-cell preprocessing). Louvain/Seurat has significantly higher ARI with ground truth across all datasets (32% *p* < e − 96, with NMI showing only 4% difference, possibly due to small number of classes vs number of cells, Fig. S6). Seurat consistently produced solutions producing higher modularity (median difference 14%, *p* < e − 148) and SE2 solutions producing higher modularity density solutions (median difference 26%, *p* < e − 56). Performance varies by dataset, for both SE2 and Louvain/Seurat (*p* < e − 30). Correspondence to ground truth was highly dataset dependent: 3 datasets averaged ~ 10% difference between the methods, while on 4 datasets, SE2 correspondence by ARI was only ~ 50% of Seurat (Additional File 1: Fig S7). Overall, these gold standard and modularity results highlight the difficulty in declaring a universally top approach to single-cell clustering. If the study goal is to mirror the style of classes that have previously been found, then Seurat is a good choice; in contrast, if the application would benefit from discovery of more granular cellular classes, SE2 will more likely produce that type of result (see Supplement on multiscale results).

The general performance comparisons above mask significant variation in modularity and ground truth in specific applications. Both SE2 and Louvain/Seurat performance (ARI with ground truth) were systematically affected by preprocessing parameters (R = 0.39, R = 0.28) with both methods being most sensitive to data reduction type (UMAP vs ICA, Additional File 1: Fig. S8). Seurat/Louvain is also very sensitive to the number of components in the reduction and number of neighboring cells. The pattern of ARI across all versions of all datasets is similar (R = 0.55, *p* < e − 82). That similarity could be driven by similar responses to datasets, as opposed to more reproducible/interpretable preprocessing parameters. Yet, even within datasets, SE2 and Louvain/Seurat had ARI’s with the ground truth that were significantly (min *p* < 0.0005) correlated, indicating they were responding similarly to preprocessing parameters (in terms of ARI with ground truth).

**Fig. 6:**
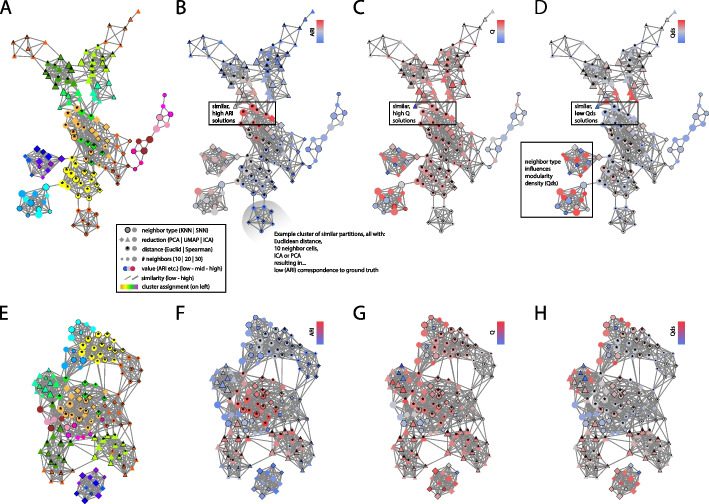
Similarity of partitions for multiple single-cell preprocessing parameters. **A** Each node represents a putative cell-type partition output by SE2, derived from unique preprocessing parameters (see legend). Edges between partitions show similarity of partitions with given preprocessing parameters, averaged over seven datasets. **B** Low (blue), medium (gray), and high (red) ARI values with gold-standard cell types for each dataset visualized. Edge thickness is proportional to partition similarity. **C** Classic/”Newman’s” modularity (*Q*) for each cell–cell similarity matrix derived from the SE2 partition of a dataset generated with a particular set of preprocessing parameters. **D** Modularity density (Qds) for each cell–cell similarity matrix derived from the SE2 partition of a dataset generated with a particular set of preprocessing parameters. **E** For a single brain-based single-cell dataset [70], SE2 is applied to data preprocessed with 144 distinct settings. **F** ARI with ground truth cell types. **G** Classic/”Newman’s” modularity (Q) of input adjacency matrix of cell similarities. **H** Modularity density (Qds) of input adjacency matrix of cells

To display trends in how preprocessing influences clustering, we generate plots in which each node represents a pool of datasets all preprocessed in a unique manner (for instance, a single node is comprised of several datasets clustered via Euclidean distance with 10 nearest UMAP-based neighbors, etc.). Linked nodes indicate preprocessing parameters resulting in similar partitions, and clusters of nodes/parameters are color coded (Fig. 6a). These networks for SE2 and Louvain/Seurat are similar in structure (R = 0.799, *p* < e − 16), indicating they are affected similarly by preprocessing options (preprocessing sets resulting in similar partitions shown in Fig. 6a). Optimal preprocessing depends on the definition of good clustering, be it ARI (Fig. 6b), modularity (Fig. 6c), and modularity density (Fig. 6d). We also show results for the individual dataset in which SE2 and Louvain/ Seurat were most similar [70]. While modularity (Fig. 6c,g) and modularity density (Fig. 6d,h) behave similarly as a function of preprocessing parameters (*p* < e − 12), the parameter setting which optimize those quantities do not optimize ground truth recovery (*p* = 0.24). These results indicate that clustering is substantially dependent on manually set parameters (distance metric, knn neighbors, resolution). This stands in contrast to the common perception of the output of Louvain/Seurat, given to the verbiage around “optimizing modularity”, might seem to guarantee robustness to a causal user.

### Finding protein complexes in high-throughput protein interaction networks

Protein binding is a core mechanism for signal propagation in cells, which makes compiling lists of proteins that assemble into complexes useful for defining functions within a cell. Specifically, treating these lists of binary protein interactions as a network, then clustering that network can group proteins into complexes that possibly have some coherent function [73, 74]. Defining a comparison partition of true protein complexes has nuances that are generally ignored, as each source of evidence for protein interactions has its own limitations and protein–protein interactions (PPI’s) contain significant noise [75–77]. Yeast proteins have been studied the longest and are provided by some of the most definitive assays, but even for yeast, there exist multiple “gold standard” definitions of protein complexes. In light of this, to provide robust results, we examine the correspondence of multiple gold standards to the clusters (complexes) produced by applying clustering algorithms to networks that are compiled from lists of PPIs. To do this, for each source of protein interactions, we first subset the network to those proteins found in the gold standard, and then we cluster those proteins with multiple methods. Furthermore, some input PPI edge lists have a confidence assigned to the interaction, so when those are available, we input them in both weighted and binary format.

An unusual feature of protein interaction datasets, relative to other domains of clustering, is that individual proteins may be assigned to multiple complexes. For instance, in the three ground truth definition of protein complexes, a widely varying fraction of proteins are assigned to two or more complexes (i.e., assigned to multiple clusters): 13% of the CYC2008 “ground truth” protein [78] were in multiple clusters, 42% in SGD [79], and 69% in MIPS [80]. This is likely due both to underlying biology, and also the manner in which protein data is compiled from multiple sources. Since SE, SE2, and Infomap can provide overlapping cluster output, we compare those methods to each of the ground truth definitions, using overlapping versions of NMI and ARI (OmegaIndex). We examine three protein networks (“Collins”, “Gavin”, “Krogan”) commonly used to test clustering algorithms [81–83], separately considering their weighted and unweighted versions, each in light of three ground truth definitions of protein complexes, for a total of 18 protein network tests.

Recovery of gold-standard protein complexes demonstrates the importance of metric selection in perceived performance of clustering methods (Additional File 3: Table 1). Evaluating overlapping clusters vs. the overlapping cluster ground truth, top performance in terms of NMI is split between SE2 and Infomap, with a 6% average percent difference in favor of SE2. In contrast, by omega index, SE2 is almost always the top performer, with an average difference of 18% over next-best SE.

Since various biases and limitations may be found in the gold-standard lists of protein complexes, we also measure the abstract quality of the clusters produced by each measure using extended versions of modularity and modularity density that are designed for overlapping community detection [84]. We test the cluster quality of both overlapping and disjoint cluster outputs, which allows us to include Louvain in the disjoint output comparison. SE or Infomap provide the highest overlapping modularity output (on average a 6% percent difference vs. SE2), while SE2 universally provides the highest modularity density (on average 22% vs. next-best SE). When all methods are forced to provide disjoint output, Louvain shows the highest modularity performance (17% difference over SE2). In fact, SE2 would have come in last in average modularity performance, except that Infomap records exceptionally low performance on two PPIs, continuing its trend of generally strong performance, interspersed with total collapse. Overlapping modularity density showed almost the complete opposite trend (as compared to classic Newman’s modularity), with SE2 showing top performance, with a 28% difference over SE, and 36% difference compared to Louvain. In summary, SE2 shows the strongest relationship with ground truth and correspondence with modularity density, while other methods are more strongly aligned with classic modularity.

**Fig. 7:**
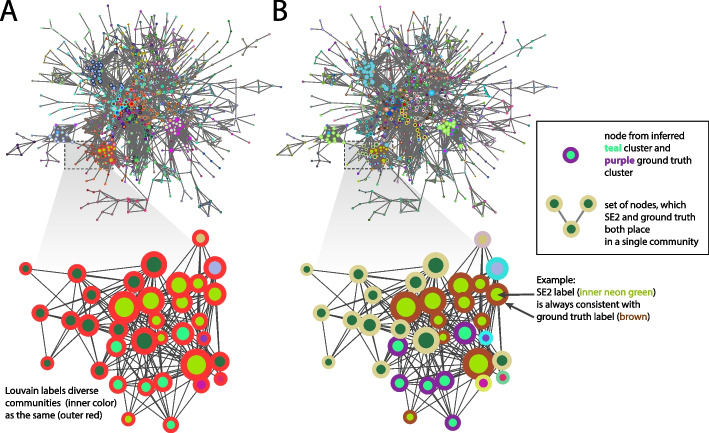
Contrast of clustering output and quality metrics in the context of protein interaction networks. **A** Cluster from Louvain, applied to protein interaction network from the Krogan data, consisting of nodes included in SGD gold-standard complexes. **B** Clusters from SE2, processing the same Krogan data. The border around each node color-codes gold-standard communities and inner color-codes SE2 communities. Inset area displays the congruence between gold-standard complexes and SE2 communities (Inner node color consistently matched with the same border color)

We provide a visually intuitive presentation of the divergence between modularity and gold standards in the performance of different methods on PPIs (Fig. 7). This visual presentation may reinforce how these differences play out in concrete terms, complementing the prior statistical analysis. Taking the Krogan PPI as an example, at first glance, the clusters detected by Louvain (Fig. 7a) generally fit the basic concept of “good” clusters, as they appear to segment the network in a reasonable manner. In contrast, the SE2 clusters (Fig. 7b) are more granular, consistent with their higher modularity density (Qds) scores. The figure inset zooms in to the scale where it is possible to make a node-by-node comparison of SE2 clusters to the ground truth. There is a strong correspondence of SE with the ground truth (inner and outer node color-coding is consistent in figure inset, indicating that SE2 and ground truth agree on clusters), while Louvain lumps multiple protein complexes into the same cluster. This indicates that there may be another aspect of community structure in protein networks, possibly corresponding to the “surprise” [35] type of node assignment utilized in SE2 (Fig. 1b), which in this example (Fig. 7a) diverges from classic modularity and even modularity density. Of course it might be possible—if they ground truth were already known—to find a resolution parameter value which better recapitulate them in Louvain. In this example, SE2 does not seem to have such circular requirements, so we also it may intrinsically focus on a particular resolution with simulation results on multiscale networks (see supplement). This single, zoomed-in example of the correspondence of SE2 with gold standards, as opposed to the modularity-optimized solution, highlights a broader divergence between the various gold standards and cluster quality metric, and between NMI and OmegaIndex (ARI). For instance, in terms of Omega Index, Louvain is generally the highest performer according to the MIPS gold standard. Therefore, while SE2 is generally the strongest performer by most metrics, caution is needed when interpreting clusters from PPIs, as the results are highly dependent on the particular network, metric, or gold standard.

### Application to diverse network classes for generalizability and runtime results

To test the performance of SE2 on an even more diverse set of networks, we utilize ~ 400 networks of several distinct origins. While these networks have relatively low membership (median: 446), the rationale for exploring partitions for these networks is to better assess how SE2 performance on synthetic networks will generalize to new applications. This assessment may be useful in determining if the method does particularly well or poorly or particular data types, but more broadly it also provides an estimate of the variability in clustering expected in the broad population of all network applications.

**Fig. 8:**
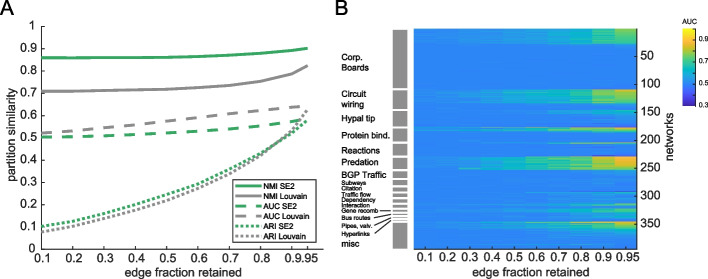
Link prediction to quantify robustness of clusters in diverse networks. **A** Various measures of cluster recovery as a function of the fraction of edges deleted from hundreds of networks in various classes. **B** The AUC for original cluster recovery for SE2 displayed for each test network, grouped into classes, sorted by AUC within class

To make results on these networks comparable to many other methods, we follow a methodology [85] that focuses on link prediction, which essentially asks to what extent clusters from subsampled data (in which a fraction of edges have been deleted) predict clusters in the full data set. In addition to this metric (reported in terms of AUC), we also show how ARI and NMI vary when comparing partitions from networks with links removed vs. partitions from the complete networks (Fig. 8a). All of these measures provide an estimate of the variability researchers can expect as they accrue data. They also provide a comparison of SE2 vs. modularity maximization (Louvain) and all the methods previously tested on this network corpus.

We find that performance (AUC for link prediction) varies as a function of network origin (*p* < 0.05), possibly indicating that some specific features of each data source lend them to be more or less stable. This can be seen in Fig. 8b, in the case of predation networks, which are highly stable/predictable even when subsampled. Stable clusters are not universal to particular classes of networks, as can be seen in the results for networks of corporate board membership. Accordingly, we find that the number of nodes, edges, and in particular edge density (*p* < e − 11) are strong predictors of performance, indicating some general network features may lead to overarching trends in clustering results. The performance of SE2 and Louvain across all subsampled networks (*n* = 3940) is highly correlated (*p* < e − 16) and did not significantly differ in performance as a function of network class. However, there are differences in the sensitivity of cluster stability metrics (AUC vs. NMI vs. ARI), and the top-ranked method also varies by metric, indicating the importance of measuring performance in multiple ways.

To test the scalability of SE2, we apply it to a range of fully connected gene correlation networks (up to 30 k nodes) as well as several technological and social networks (10 m nodes) [86]. The very largest networks tend to stem from technological sources, and thus results may not be indicative of all future applications of clustering. SE2 is 60% faster the original SE and several times faster than Louvain and Leiden, on fully connected networks (Additional File 1: Fig. S9). It is also several times faster than WGCNA, which was specifically designed to operate on such inputs. However, Louvain and Leiden are orders of magnitude faster than SE2 in clustering large sparse networks (Additional File 4: Table 2). For details of performance testing and significant caveats to interpreting these, see Supplementary methods. In terms of the quality of results on large networks, the modularity-optimizing Leiden returns modularity values with mean 16% difference over SE2. In terms of modularity, SE2 has a mean 35% difference over the next-best SE and 135% over Louvain. Infomap appears to falter in this setting, providing low modularity and returning most nodes in a single cluster (Additional File 4: Table 2).

## Discussion

Each year, thousands of studies in biology utilize clustering methods, to provide an overview of data trends. While this approach is intended to provide robust results, those clusters can be unstable and suboptimal, as demonstrated by the results on thousands of networks shown here. As we have observed, performance can vary based on the specific application, with popular methods sometimes failing unexpectedly and severely. Competing definitions of good clusters further lend additional complexity to the quest for the best method. These challenges to robust clustering likely affect the many studies that utilize clustering, reducing their reproducibility. To redress this situation, hundreds of new variations on clustering methods have been proposed [87], each of which typically report superior results on a small set of networks. Such methods are almost never tested on biological data, while methods coming from biology labs are typically only tested on very specific data types. Furthermore, results on few datasets and specific data types cannot support claims for universal superiority, when theoretical results and those observed here indicate there is no universally optimal method for all applications.

In light of these practical limitations and large-scale problems in the field of clustering method, our approach has been to assemble a large and diverse group of networks and metrics to plausibly survey situations in which SE2 and other methods may be useful. If we were to only select a single one of these applications or parameter sets — which is the typical approach in the field — it could easily present a warped view of the overall performance of the methods. We also investigated important practical aspects of clustering — ability to produce meaningful clusters, ability to process negative edges, ability to produce overlapping output, and ability to process massive networks — in addition to the classical question of accuracy.

Selecting a clustering method is not independent of the intended application. For instance, if there were some a priori reason to suggest that classic modularity is indeed the appropriate definition for the clusters represented in your data, then Louvain/Leiden is likely the optimal method to use. However, it is often utilized when the number of communities is unknown, in which case it is open to bias and results between studies may be less consistent. Arguably, different methods without resolution limits, or methods that attempt to optimize metrics without resolution limits [17, 22], could be superior to Louvain and SE2 in a specific context. At the same time, in protein networks, we have seen instances where the highest correspondence to ground truth clusters is neither modularity nor resolution-free metrics, indicating there are not universal suggestions for the optimal method and metric across all datasets. One concurrent explanation for SE2’s success outside of classic metrics and across many applications is that many networks have clusters detectable on the basis of “surprise” [35], or the unexpected of a partition, which in our case means grouping nodes with the most unexpected common number of labels among their neighbors. All of the changes to SE2 vs. the original SE are consistent with this idea, and the changes essentially help SE2 to do a more thorough job of computing surprise by enacting it on multiple scales and avoiding local minima. In addition to selecting a clustering algorithm on the basis of performance, questions of feasibility/run time on big biological data can also rule out certain methods. Here, SE2’s ability to relatively quickly process fully connected networks can be useful, as many correlation-based networks in biology are in this format.

### Critiques and limitations of SE2

Historically, development of clustering algorithms has largely been driven by the search for more accurate cluster recovery. SE2 and Louvain both return clusters that have higher modularity than the ground truth, suggesting a turning point where issues of reliability and scalability are relatively more challenging. Currently, the pool of partitions only serves to increase robustness (SE2 selects the average partition) and to assist in finding multi-community nodes (whose labels do not strongly select a single community in any partition). However, this pool of partitions could serve to either maximize a clustering quality metric (like modularity) or to iterate towards a more stable label solution. For instance, SE2 could take higher-Q partitions as the initial labels for a subsequent run to potentially optimize modularity. To improve the fit of labels, SE2 could take a mixture of prior solutions as the input to subsequent runs, which could help it to ratchet closer to the most optimal solution. Because the source of SE2’s improvement over the original SE are the additional ways SE2 updates labels at the level of communities (see supplement), as opposed to individual nodes, further label update modifications could also increase accuracy.

While there is room to improve the accuracy of SE2, results on synthetic benchmarks indicate that accuracy of this method and some others is approaching the limits of testability, as test networks are so noisy that incorrect clusters have higher modularity than the original clusters. Moreover, it is important to specify exactly which metric is used for accuracy, as there are likely tradeoffs for improving one metric at the expense of another. Furthermore, while improvements in accuracy are possible, scalability will likely become a more distinguishing feature, as the number of variables in omic and multi-omic technologies grows. The most time-intensive step in SE2 is aggregating the counts of labels to which each node is connected. To improve scalability, we have limited the cost of this step by reducing the number of unique labels present in the network at any time, compared to the original SE. Since limiting the number of clusters improves execution time, an alternative cluster splitting mechanism could improve results: for instance, the Fiedler vector has been used to split clusters, however, we did not find any improvement in overall results compared to randomly splitting clusters. On very large networks, performance of SE2 is most likely to be memory limited, since each processor needs access to the full adjacency matrix, and the current parallel implementation entails making copies of the adjacency matrix for each independent run. Addressing this limitation could improve runtime and memory usage by an order of magnitude, but would require integrating sparse memory mapping libraries in a low-level language, which is in progress [88].

## Conclusions

In light of the limitations of all clustering methods and metrics, the practical question of how to cluster data comes down to inter-related issues of (1) accurate performance amid high noise, (2) robust cluster identification on new data, and (3) reliability of the method itself. By testing several clustering algorithms on major classes of biological network data, synthetic networks, and even networks outside of biology, we attempt to determine where they will function best, and to infer the probability they will be useful on novel types of biological data. In surveying the performance of SE2, we find that it is useful to employ multiple metrics: for instance, both comparisons to ground truth and abstract measures of cluster quality. Finally, we show that performance can vary widely due to the features of specific datasets, implying that applications across thousands of networks, as we have performed, are necessary for a global understanding of performance. In summary, the future of evaluating clustering methods in a meaningful way is a difficult task that goes beyond demonstration in a few networks. While we observe that SE2 can provide top performance in specific applications, the more useful result is its acceptable performance across diverse applications and performance metrics. This suggests, but does not guarantee, that it may be useful in searching for robust clusters across datasets or in new data types.

## Methods

### The overarching goal of SpeakEasy 2

Champagne is to identify clusters by maximizing the specificity of the labels assigned to nodes, where the specificity of a node’s label is defined as the difference between the actual frequency of that label in neighboring nodes compared to the expected frequency, given the global popularity of each label (pseudocode in Additional File 5, [89]). In the process of attempting to maximize this quantity across all nodes, SE2 alternates among four types of activity, some of which increase the number of labels. In contrast, most label propagation algorithms have a single mode of activity, during which node labels are updated, and the number of unique labels constantly decreases as spurious labels are weeded out. After looping through these four modes of activity, SE2 records the label state as a possible partition (Fig. 1H, Additional File 6). This process is repeated (default = 10x), starting from independent initial labels to generate a pool of partitions, from which the final partition and any multi-community nodes are selected.

### Initialization

For the first time-step, SE2 overloads labels, with the number of unique labels set to 1% of the number of nodes. Rationales for limiting the number of labels (as opposed to assigning a unique label to each node) include the following: reduced memory usage, faster convergence—which potentially increases accuracy as more runs can be accomplished, and more efficient computation—as most time is spent on relevant label updates as opposed to labels that ultimately die out. The ratio of self-connections (main diagonal of the adjacency matrix) to the total non-self weights affects the propensity of nodes to join other communities versus remaining isolated. Therefore, when the average skew of the edge weights is high (< 2), we set the self-connection weight to the average edge weight for that node, and non-self edge weights are unchanged. If the maximum weight in the input adjacency matrix is less than one, they are rescaled so the maximum value equals one; otherwise, weights in the input adjacency matrix are unadjusted, as long as they are in the range of [− 1 1] and not heavily skewed as described above.

### Standard label update stage

This function is the most commonly applied step and similar to the classic SpeakEasy operation. In this step, each node adopts the label that is most specific to its neighbors. This selection is weighted by the edges to those neighbors, taking into account the global frequencies of those labels. Unlike the classic version, we do not aggregate information from several previous time-steps, which was intended to stabilize label evolution. Due to the new bubbling and merging stages, we only need to use the most recent label information (i.e., from the previous time step), which improves runtime. At each time-step, 90% of the labels are updated, which avoids oscillating solutions in certain small binary networks.

### Bubble stage

It is possible that subsets of nodes within an existing cluster would be more optimally positioned in distinct clusters, but that the subsets will never achieve this more optimal configuration due to the gradual nature of single-node updating. Essentially, some partitions can only be reached through concerted action on a group of nodes. Therefore, we implement a destructive “bubbling” stage that disperses nodes with the least secure labels within large clusters. Other clustering algorithms have attempted non-random splits among clusters. While it is logical to attempt to preserve high-quality existing structures/existing subclusters, we did not find an improvement in performance on LFR versus random splitting, which also incurs minimal overhead. The overall goal of the merging and bubbling stages is to expose nodes to a wider range of potential labels than they would experience with single-node updating, while still allowing the system to converge.

### Nurture stage

One aid in convergence is attempting to preserve proto-clusters with good fit, produced during the bubbling stage. To do this, we perform selective label updating only on nodes with relatively poor label fit — i.e., those nodes whose neighbors do not suggest a definitive label. This prevents more promising label configurations from being wiped out prematurely, as those remain in place and lower-confidence labels organize around them.

### Merge stage

While most label propagation algorithms update nodes individually, during this stage, we perform label-wide updates, which allow us to more quickly reach a maximum label specificity. Specifically, the pair of labels with the highest frequency of cross-linking are merged into a common label. This process repeats until no label has greater-than-expected cross-linking with any other label, where expectation is defined as the number of connections that would be between two labels if all connections were random in the network. The benefit/goal of this group-wise updating is that, at the onset, while each individual node has adopted its own optimal label, the set of nodes will never achieve an even more optimal state without this collective (label-wide) action.

### Termination and consensus clustering stage

Partitions are sampled after the algorithm has passed through all four stages several times (default = 5). Passing through the stages generates partitions that are largely independent of each other, but there may be some historical influence. Therefore, the default behavior is to gather solutions from a number (user determined, default = 10) of completely independent runs as well. The final partition selected is that with the highest NMI with all other partitions. Thus, this final partition is a very typical partition, as opposed to optimization approaches that might select the partition with highest modularity.

To confirm the benefit of each of these new updating strategies, we compared performance with and without each new type of label updating step, on a wide range of LFR networks (see supplement for details). In summary, all of the new updating functions resulted in significant improvements with the greatest contribution due to the cluster splitting step (see supplement).

### Multi-community node identification

Evidence for multi-community nodes is derived from the complete set of partitions and all possible label assignments. Specifically, if there is only a small difference in the score of the best community for a node and the second-best assignment, and this is consistently the case across many partitions, that node will be selected as multi-community. Note, the selection of multi-community nodes is performed independently of their assignment to specific communities. For instance, if a node is determined to be a member of three communities (based on all partitions), it will be placed into the three communities with the highest scores (most unexpected labels) recorded in the final partition.

### LFR network pool

We generate LFR test networks for all possible combinations of all parameters in the method, for a range of values that spans those typically seen in publications utilizing LFR networks. The parameter space of the networks contains the following: number of nodes = [2000 | 20000]; average degree [30 | 60]; cross-linking weights (*μ*_w_) [ 0.1:0.2:0.9 | set to match *μ*]; cross-linked edge fraction (*μ*) [0.1:0.2:0.9]; community size distribution exponent [1.5 | 3], weight distribution exponent [1.5 | 3]; degree distribution exponent [1.5 | 3]. When the maximum off-diagonal edge weights from LFR is < 1, we rescale all edges so the maximum weight = 1. Networks are also generated under three different regimes, where the cross-links between clusters (1) have covarying proportion (Fig. 2) and weights, (2) constant proportion and varying weight (Additional File 1: Fig S2), or (3) varying proportion and constant weight (Additional File 1: Fig S3).

### Origin of single-cell datasets

All 7 datasets associated with gold-standard cell types have uniform QC [90]. We then generate cell–cell similarity matrices with all possible combinations of the following parameter settings: gene expression variance cutoffs [top 15 | 30%]; number of connected/neighboring cells [10 | 30 | 50]; proximity measure [Euclidean | Spearman]; number of principal components [5 | 20]; data reduction type [PCA | ICA | UMAP]; neighbor selection type [KNN | SNN]. These dimension-reduced adjacency matrices are then provided to SE2 and Louvain (essentially to emulate Seurat, while providing more direct preprocessing control).

### Supplementary Information

**Additional file 1.** Supplementary text and supplementary figures.

**Additional file 2.** Movie of operation of SE2 on demonstration network.

**Additional file 3: Table 1.** on PPI networks.

**Additional file 4: Table 2.** on large technological networks.

**Additional file 5.** SE2 Algorithm Pseudocode.

**Additional file 6.** Global flowchart of all SE2 operations.

**Additional file 7.** Review history.

## Data Availability

Code availability: The MATLAB version of the code is freely available under the MIT license at github.com/cogdishion/SE2 [89] and zenodo [91] https://doi.org/10.5281/zenodo.8322854). The C version is available under the GPLv2 + license at github.com/DavidRConnell/igraph-SE2 [92] and zenodo [93]. The datasets generated during and/or analyzed during the current study are available via: https://www.radc.rush.edu/requests.htm (ROSMAP gene expression), https://www.synapse.org/#!Synapse:syn30821562 (Mayo and Mt. Sinai gene expression), https://github.com/mkrzak/Benchmarking_Clustering_Methods_scRNAseq (single-cell data) and https://paccanarolab.org/cluster-one (protein binding), https://github.com/Aghasemian/CommunityFitNet (diverse network set) and https://snap.stanford.edu/data (large networks).
