# Supplementary material for: Robust, scalable, and informative clustering for diverse biological networks
Source: Genome Biol. 2023 Oct 12;24:228. doi: 10.1186/s13059-023-03062-0 (PMC10571258; doi:10.1186/s13059-023-03062-0)

**Supplementary Information**

**Benefit of each type of label updating to SE2 performance**

The major changes in SE2 compared to the original are the addition of three new types of node label updating steps. To specifically demonstrate and quantify their contributions to performance, we examine the effects of deleting each type of label update. The network test set for this is all prior LFR networks with mu=0.7, chosen to largely avoids floor and ceiling effects. Performance is measured in terms of ARI, which show larger variability than modularity, as the point is to understand the relative benefits of each type of updating. The greatest effect on performance was produced by eliminating the cluster splitting step, resulting in an 83% decrease in ARI in our specific test set of networks. Eliminating the higher-level merging step decreased ARI by 33% and eliminating the nurturing step that occurs after splitting reduced performance by 16%. There are some limitations to this testing – the nurture phase is invoked after splitting, so when splitting is removed, there is also no nurture step. Performance (NMI) may actually be reduced to below that of the original SE, because SE2 may initialize with too-few labels for the true number of clusters, and if there is never the opportunity to split those, it will be impossible to meet the true number of clusters. All of this may lead to over-emphasizing the importance of cluster splitting, though it may still be the leading contributor.

**Interpretation of LFR testing under high levels of cluster cross-linking**

Studies utilizing the LFR networks typically co-vary both the number of cross-linking edges and the weight of those edges, likely as results of this approach are the easiest to interpret. However, other approaches have also been used, showing the effect of solely varying the weight of cross-linking edges, with a constant moderate amount of cross-linking edges. Therefore, we also test that scenario, in which the number of edges that cross-link different clusters is set to a moderate constant, and their weight is varied. Performance trends across methods for this approach are similar to Figure 2 (showing covarying weights and cross-linking edge number), as increasing cross-linking weight results in less accurate cluster detection, as per NMI and ARI (Additional File 1: Fig. S4ab). At the highest levels of cross-linking, it is also possible for SE2 and Louvain to detect clusters with higher modularity (values greater than zeros in Additional File 1: Figure S4d) than the original LFR cluster definitions. Modularity density shows similar trends as modularity, sans artifacts at the highest levels of cross-linking, which originate as shown for Additional File 1: Figure S1.

For completeness, we also test the converse of the above approach - increasing the frequency of cross-linking edges with a constant weight assigned to them. In this framework, as μ increases, the ability of most methods to recover clusters is non-linear (U-shaped performance in Additional File 1: Fig. S3). This arises because, at low μ-values, the few existing cross-linking edges must carry the entire (relatively high) cross-linking weight. This situation makes it difficult for several methods to detect true clusters, with SE2 showing significantly higher performance at this level (p<e-6). At high levels of cross-linking, cluster recovery is actually easier than the case wherein cross-linking and the weight of those cross-linked edges are paired. In this class of network tests, the top performer shifted from SE2 at low μ, to either Infomap, or Louvain showing top performance at high μ. Significant differences between methods is rare, with narrow improvements in any method over SE2 (p<.05) in NMI or ARI, at high μ-values. Infomap performance shows top performance in terms of NMI for cluster recovery (Additional File 1: Fig. S3a) and ARI (Additional File 1: Fig. S3b), in contrast to its near-last performance in the alternate slate of networks, wherein cross-linking weight was matched with μ. The basis for this shift is unclear, but it demonstrates how radical shifts in perceived performance can result from selective benchmarking. In this case, even when running hundreds of networks, the shift in Infomap performance was only seen when we tested this uncommon constant cross-linking weight setting.

We note a caveat for the interpretation of modularity density values (Qds) values. Because modularity values become close to zero for high μ-values, and because they may exceed the modularity of the original LFR solution, we display them relative to the original LFR solution value (Additional File 1: Figure 2H). These values might seem to support the validity of Infomap and NMF at the highest μ-values, despite their low performance by all measures at low μ-values. The origin of this appears to be that modularity density produces very small, but non-zero, values when all nodes are placed into a single cluster (Additional File 1: Fig. S1). This minor artifact only surfaces in these extreme scenarios, wherein Infomap and NMF are essentially failing by lumping all nodes into a single cluster, which occurs in all three LFR scenarios. Further indications of this is that percent change in Qds relative to the Qds value for the LFR solutions is correlated with the maximum cluster size for NMF and Infomap (p<e-14), but that is not the case for SE2 p=0.46.

While performance of the non-negative matrix factorization (NMF) algorithm was almost never competitive, we find an additional limitation for that method. Specifically, NMF required either excessive run times (>week) or out of memory errors (>512GB) on large LFR networks (20,000 nodes), so all results for that method are from 2000-nodes networks. Intuitively, this would indicate that the reported NMF results are better than reality. While that may be the case, the performance of other methods did not drop significantly for larger networks. Since NMF is already generally the lowest performing method, this limitation likely does not meaningfully affect interpretation of results.

**Additional comparisons on networks from bulk gene expression**

The ideal partition from transcriptome data would segment all biological processes that are active in a tissue into distinct modules. The extent to which this occurs is practically measured by tabulating the number and strength of GO terms that are over-represented in any module.

To compare SE2 and WGCNA in this setting, for all GO terms that are significant in either method, we compare their significance in the alternate data set. The overall significance of the categories detected by both methods was highly correlated (R=0.95 p<e-240) indicating they agree on the relative involvement of various molecular systems in generating coexpression modules. Under a threshold of Bonferroni significance p>.00005, 85% of categories from an individual method were significant in both, and the top enrichment for each module was always also significantly detected under the alternate method. Thus, from a functional enrichment perspective, the set of biological functions detected in this dataset are highly similar in these methods. However, there are still differences in the gene composition of each module (as seen from NMI and ARI results) that may affect the average signals from those modules. Any such differences may be important as they will affect module-trait associations, which is the typical end point for coexpression analysis and clustering.

WGCNA has the unusual issue of often placing thousands of gene-nodes into an unclassified group, which is not formally a module. This causes some issues for interpreting results of figure 5, which we explain in detail here. While NMI and ARI often have differences in magnitude, their trends are generally similar, so we investigate these opposing results and find they are due to modules with very large membership in WGCNA. The largest module found by WGCNA across all sample sizes and replicate runs contains 3386 genes, which is 3.3 times the size of the largest average module size produced by SE2 - such large modules can exert a strong influence on ARI. WGCNA also outputs another very large gene set that is treated as a module, but which consists of genes that are not placed into any group - on average 2349 genes are in this unassigned group, across all replications. Such un-assigned genes can be problematic for biological interpretation, so an extension with 22 manually-tuned parameters was released, in part, to address this issue (). To directly test if these very large modules drive opposing NMI and ARI results, we remove the unassigned genes and genes in the largest module from consideration. After these changes, ARI between small sample size partitions and the held-out partition is also higher in SE2 (by 16%, p<e-5), with NMI remaining significantly higher in SE2. Thus, the unassigned genes (which are grouped in a pseudo-module) and the one relatively large module account for the divergent ARI results, consistent with ARI’s emphasis on the size of cluster overlaps.

To provide replication for these results, we utilize the next-largest brain-based bulk RNAseq data is from the Mt. Sinai (frontal pole) and Mayo brain banks (temporal cortex). Due to the smaller sample size, we use are resampling approach to understand the stability and quality of coexpressed gene clusters generated by SE2 or WGCNA. Specifically, we resample the data with replacement, then cluster each with each method 100 times. We find that the stability of SE2 partitions is significantly higher – mean NMI between all partitions for the Mt. Sinai frontal pole data is .93 (vs .44 for WGCNA, p<e-16) and mean ARI .95 (vs .48 for WGCNA, p<e-16); mean NMI between all partitions for the Mayo temporal cortex data is .92 (vs .43 for WGCNA ,p<e-16) and mean ARI of .92 (vs .37 for WGCNA, p<e-16). In practice, these results indicate the SE2 is less likely to swap genes between clusters on replicate datasets, which should benefit data/cluster replication over time.

However, these results do not rule out the possibility that SE2 is consistently stuck in a suboptimal state. Addressing that possibility, mean modularity of all partitions in the Mt. Sinai data for SE2 was .085 (vs .049 for WGCNA p<e-16), and for Mayo SE2 was .20 (vs .073 for WGCNA, p<e-16), indicating that SE2 output is not only more stable, but also of higher quality (at least in terms of modularity). Results described are for one level of subclustering in SE2, as this can be required for some RNAseq datasets, and this produced a number of clusters most similar to WGCNA. Less subclustering produces higher modularity (but overly broad clusters from a gene ontology perspective) while further subclustering (i.e. sub-subclustering) produces lower modularity-values, so these intermediate results from SE2 appear appropriate.

**Origins of “gold-standard” single cell clusters**

To place the gold-standard comparisons in context, we review the origins of each gold standard and find that the ones using classic definitions of cells may be the least aligned with gene expression, while others utilize some element of clustering to define the partition. For instance, some studies (66,67) note rough visual correspondence between inferred clusters and some biological state (cell cycle or embryonic development days), but it is also clear that classic divisions are not always aligned with gene expression. Several cell type definitions from several datasets involve manual control over clusters, which brings up questions of robustness and circularity in those “ground truth” cell types (68-69). Other datasets have likely more robust cell type definitions. For instance, one dataset analysis (70) utilized multiple types of clustering to help ensure robust cell type definitions, and such a consensus approach is possibly the best way forward in novel data sets. Possibly for this reason, SE2 outperforms Seurat on this dataset, in terms of ARI with that ground truth.

**Applied performance testing for fully connected and sparse networks**

The goal of this comparison was to identify relative speed of execution for various clustering methods (Additional File 1: Figure S6). For the first performance comparison, we used real data (gene expression data) instead of random edges, as some algorithms may require convergence to a stable solution as a criterion for completing. To generate correlation-based networks larger than the original number of genes (~15000), we duplicate randomly selected genes with added noise. For methods which cannot process negative edges, we used the absolute value of the correlation as the edge weight. When available, we utilized code from the original authors to obtain the most accurate performance. Consequently methods are coded in several languages, which naturally influences performance vs the theoretical ideal for the method. Similarly, various method employ parallel processing to different extents, and we did not attempt to control this behavior (all methods tested on a dual 32-core EPYC server). All algorithms were run at default settings, and altering those may affect performance. Also relevant to the interpretation of results, SE2 and SE produce 50 partitions under the default settings used, while all other methods generate a single partition.

Performance results from Infomap are not practically useful because for full connectivity matrices it returns all nodes in a single cluster. The reason for SE2’s faster performance on larger networks and much slower performance (vs Louvain/Leiden) on sparse networks is likely related to the community updating function – the most computationally time-intensive step. SE2 updates the community assignment of the majority of nodes at every time step. In contrast Louvain and Leiden typically update the community assignment of nodes which have neighbors that have been recently reassigned to new clusters. This universal updating in SpeakEasy has some runtime benefits on fully connected networks (wherein Louvain must also update all nodes due to the full-connected structure) but creates inefficiencies in the sparse context (which are difficult to avoid without moving to a C implementation and selective node updating more akin to Louvain). Accordingly, in a second performance comparison we record completion time for a diverse collection of large sparse networks (Table 2). Here Leiden and Louvain are orders of magnitude faster than SE2, although SE2 runtimes are still practically feasible.

**Multiscale performance of SE2**

Because many biological networks have community structure on multiple (generally hierarchical) “scales” (levels of resolution), we investigate how SE2 responds in this setting with multiple plausible correct clustering solutions. For instance, when there are multiple plausible clustering solutions for a given network (all existing simultaneously, but at different scales/resolutions) which resolution will SE2 generally select? To form relatively robust conclusions on this topic, we generate a pool of test networks (n=144), comprised of sparse unweighted networks with realistic connectivity (exponentially degree distributions) and multi-scale hierarchical community structure on three levels. Each network will have a unique combination of parameters, including connectivity degree distribution exponents ([-1.5 -3]), node number ([5000 10000]), proportions of edges between communities on different scales ([.1 .2]) with 3 replicates having different random community split sizes between scales, all of which lead to connectivity density in the range of (0.18-1.2%). For each network, we also produce examples whose community structure is strongest on a particular scale, varying the proportion of edges in this strongest scale ([.4 .55 .7]); according modularity values were generally highest for the ground truth at the enhanced scale (R=.21, p=.01). This enhanced connectivity at specific scales is one check for bias towards/against a particular low/high resolution, and also simulates that case where maximal information on community structure may be available at a particular scale.

For partitions generated by SE2 and the Leiden algorithm on this collection of networks, we examine the scale to which they most closely correspond and their similarity to ground truth. Partitions from SE2 show the highest correspondence with the connectivity-enhanced scale in 80% of networks (p<e-5). Results from the Leiden algorithm are more complex, as it will output varied clustering solutions based on a manual input parameter, referred to as the resolution parameter. To see how Leiden optimally responds to the pool of multi-scale networks, we first tune the range of the resolution parameter range to be ideal for this application. This tuning produces three resolution values, one for each scale, which (on average for this pool of networks) induce Leiden to produce partition wherein the number of communities equals the true number of communities. Of course such information on the true number of communities is generally unknown, but this provides a high bar for comparisons with SE2. We find that when the scale-relevant resolution parameter is supplied to Leiden, it produces the highest ARI with the enhanced scale in 34% of cases, which is not significant (p=.24). Why is Leiden finding the enhanced scale less frequently than SE2, even when it has been supplied with appropriate tuning (resolution) values? A clue to this is found in that the mean ARI of Leiden with the enhanced scale is .86, while that of SE2 is .74. Thus Leiden is outputs solutions with higher correspondence to *some* ground truth, just not the level of the network that has enhanced connectivity. The scale to which Leiden’s partitions most closely correspond to (by ARI) is on average 0.8 scales higher (more coarse) than the enhanced connectivity scale, while SE2 is only -0.04 scales away from the enhanced connectivity scale. The relationships of these algorithms to the ground truth is swapped from what was observed in LFR networks, wherein SE2 typically had higher ARI with ground truth. In terms of the quality of solutions produced, modularity of Leiden partitions is on average 99.5% of that of the ground truth, while SE2 is 94%. As with prior LFR networks, modularity density of SE2 partitions is higher (by 5%) than that of Leiden partitions.

To further explore the tendency of an algorithm towards a particular scale of networks organization, we also generate another slate of 144 networks of varied characteristics, but in which connectivity is *equally* distributed across scales. The Leiden algorithms was supplied with the same range of resolution parameter values which should allow it to output partitions across a range of scales. For this pool of networks, SE2 partitions typically (78% of partitions) most strongly reflects the intermediate scale, while Leiden typically (84%) most reflects the top (most coarse) scale.

In summary, in the context of this diverse collection of sparse unweighted networks, partitions from SE2 tend to represent the scale with the clearest community structure. Even when attempting to guide Leiden to the single most ideal scale, it frequently deviates towards a more coarse scale. Louvain/Leiden do offer the opportunity to tune the resolution, so in a specific application, a user can dial down the resolution until results appear “good”, although such manual adjustment is time-consuming and of unclear robustness. A related weakness of SE2 is that it may require additional subclustering in specific contexts such as RNAseq data, which has overarching technical influences alongside biological factors, generating hierarchical multiscale community structure. However, in this more pure (but still diverse) pool of multiscale networks, SE2 displays the interesting property of being scale-sensitive, compared to Leiden. Examination of partitions for these networks also continue to demonstrate the importance of considering a range of performance metrics, as modularity, modularity density and ground-truth similarity all offer slightly different appraisals of the performance of these clustering methods.

**Complexity Analysis**

*Initialization*

# Calc k_in_ once: O(|V| + |E|) ≤ O(2|E|) = O(|E|)

# Produce initial random labels: O(|V|) per independent run for total of O(|V| * #Runs)

# Assign one core per independent run (Negligible initialization)

*Main body*

Indeterminant # time-steps, looping through 4 operations (“typical”, “nurture”, “bubble” and “fusion”), one of which will be executed at each timestep.

- Operation: “typical”. Updates node label based on difference between actual and expected labels among neighbors. Majority of time-steps are in this mode. Overall: **O(|E|)**.
  - Calc expected distribution of labels **O(|V|)**
  - Calc actual local distribution of labels **O(|V|)**

Per node **O(|V|)** calc neighbors **O(1)** then count label per neighbor **O(numNeighbors)** -> **O(|V| * avg numNeighbors)** -> **O(|E|)**

get the difference precalculated expected and actual calculated above is: **O(nLabels * |V|)** -> **O(|V|^2^)** (as labels grow linearly with V)

- Operation: “nurture”. Effectively the same as “typical” except run on a subset of nodes. **O(|E|)**
- Operation: “bubble”. This randomly assigns all members of some clusters to new random labels. **O(#Labels*|V|)**
- Operation: “fusion”. This requires calculating all cluster-cluster connectivity strengths. Reasonable assumption is there are log(V) communities, so $\mathbf{O}\left( \left( \frac{\mathbf{|V|}}{\mathbf{log|V|}} \right)^{\mathbf{2}} \right)$.

*Postprocessing of partitions*

# Representative partition selection O(#Runs * |V|log(|V|))

- pairwise NMI calculation **O(|V|log(|V|))**
  - Total then **O(numPartitions^2^ * |V|log(|V|))**

**Supplemental Figure S1. Modularity in comparison to size of largest cluster, by clustering method.** Node transparency is scaled to μ - higher opacity with higher μ. Histograms of distributions for each method on axes.

**Supplemental Figure S2. Recovery of disjoint clusters in LFR networks, constant cross-linking edge number, variable cross-linking weight.** A) Recovery of original LFR communities, quantified by normalized mutual information (NMI), grouped cluster cross-linking weight. B) Recovery of original LFR communities, quantified by adjusted rand index (ARI). C) Classic Newman’s modularity (Q) of inferred clusters. D) Comparison of modularity of inferred clusters relative to the modularity of the ground truth solution. E) Modularity density (Qds) of inferred clusters. F) Comparison of modularity density of inferred clusters to that of the ground truth solution.

**Supplemental Figure S3. Recovery of disjoint clusters in LFR networks, constant cross-linking weight, variable cross-linking edge number.**

A) Recovery of original LFR communities, quantified by normalized mutual information (NMI), grouped by levels of cluster cross-linking (“μ”, x-axis). B) Recovery of original LFR communities, quantified by adjusted rand index (ARI). C) Classic Newman’s modularity of inferred clusters. D) Comparison of modularity of inferred clusters relative to the modularity of the ground truth solution. E) Modularity density of inferred clusters. F) Comparison of modularity density of inferred clusters to that of the ground truth solution.

**Supplemental Figure S4. Recovery of overlapping clusters in LFR networks, constant cross-linking edge number, variable cross-linking weight.** A) Cluster recovery quantified at varied levels of cross-linking weights via normalized mutual information (NMI). B) Cluster recovery quantified by adjusted Rand index (ARI). C) Cluster quality measured by classic Newman’s modularity. D) Cluster quality measure by modularity density. E) Accuracy of overlapping community assignment as nodes are members of varied number of communities. F) Ability to detect overlapping nodes under increasing cross-linking. G) Ability to detect overlapping nodes, under varying levels of multi-community memberships.

**Supplemental Figure S5. Recovery of overlapping clusters in LFR networks, constant cross-linking weight, variable cross-linking edge number.** A) Cluster recovery quantified at varied frequency of cross-linking edge via normalized mutual information (NMI). B) Cluster recovery quantified by adjusted Rand index (ARI). C) Cluster quality measured by classic Newman’s modularity. D) Cluster quality measure by modularity density. E) Accuracy of overlapping community assignment as nodes are members of varied number of communities. F) Ability to detect overlapping nodes under increasing cross-linking. G) Ability to detect overlapping nodes, under varying levels of multi-community memberships.

**Supplemental Figure S6. Performance by multiple metrics, across all datasets and preprocessing parameters, for Seurat/Louvain and SE2.**

**Supplemental Figure S7. Performance by dataset, across all preprocessing parameter settings, for Seurat/Louvain and SE2.**

**Supplemental Figure S8. Multiple types of performance as a function of levels of preprocessing parameters for Seurat/Louvain and SE2.**

**Supplemental Figure S9. Runtime performance of several algorithms on fully-connected networks based on gene expression.**

**Supplemental Figure S10. Typical time course of SE2, showing different types of algorithm activity.**

**Additional File 2. Movie of labels for a small network evolving over time, showing the type of activity SE2 is engaged in at each step.**  Labels are color coded. Network layout emphasizes ground-truth clusters. Edges between nodes currently with same label are darkened.

**Additional File 3: Table 1 Recovery of gold-standard protein complexes from protein-protein interaction (PPI) networks, and modularity scores of inferred complexes.**

**Additional File 4: Table 2 Cluster quality metrics and runtime for large networks.**

**Additional File 5. Pseudocode for SpeakEasy2: Champagne.**

**Additional File 6. Detailed Flowchart of SE2 actions.**

**Figure S1**


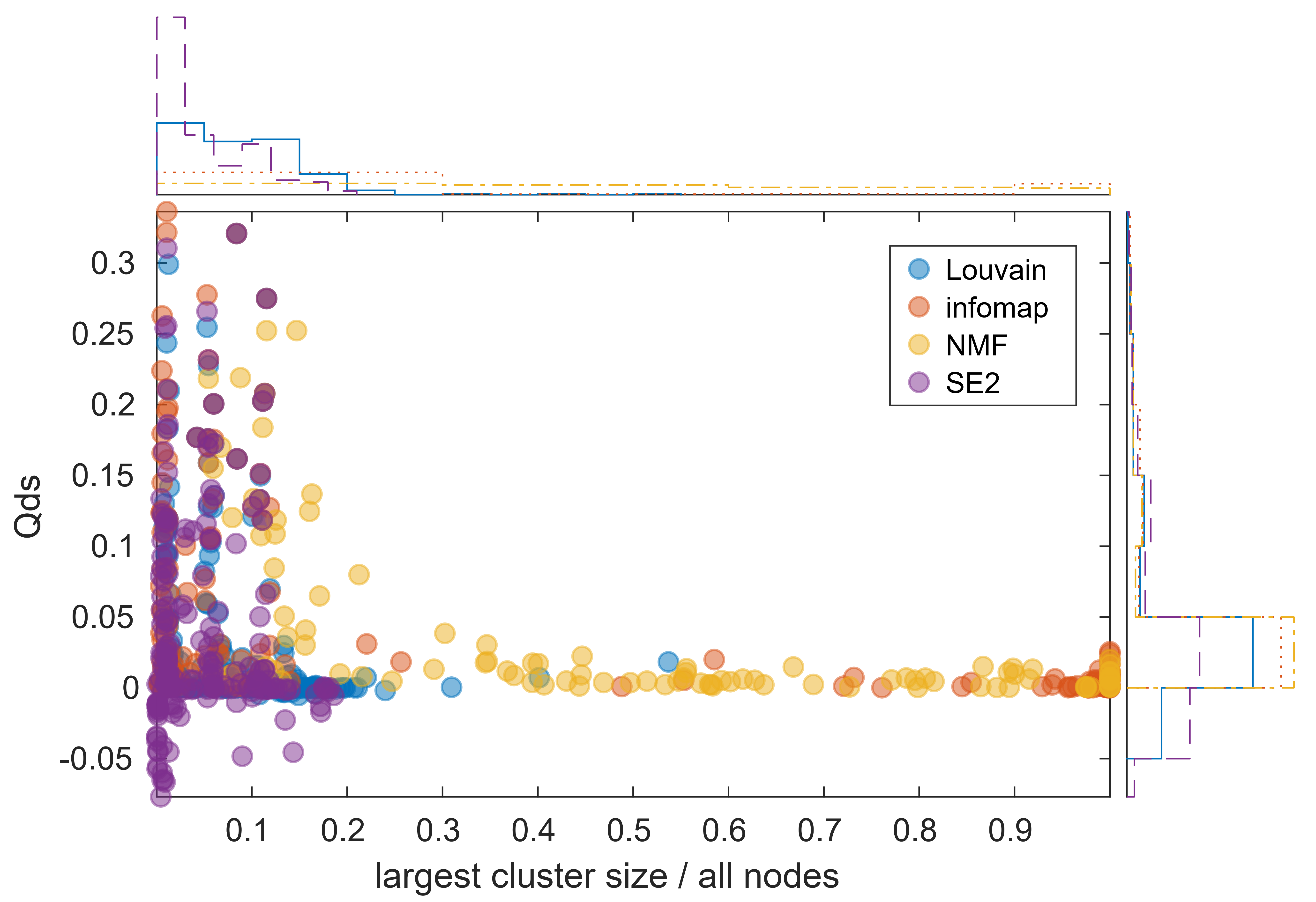


**Figure S2 (below)**


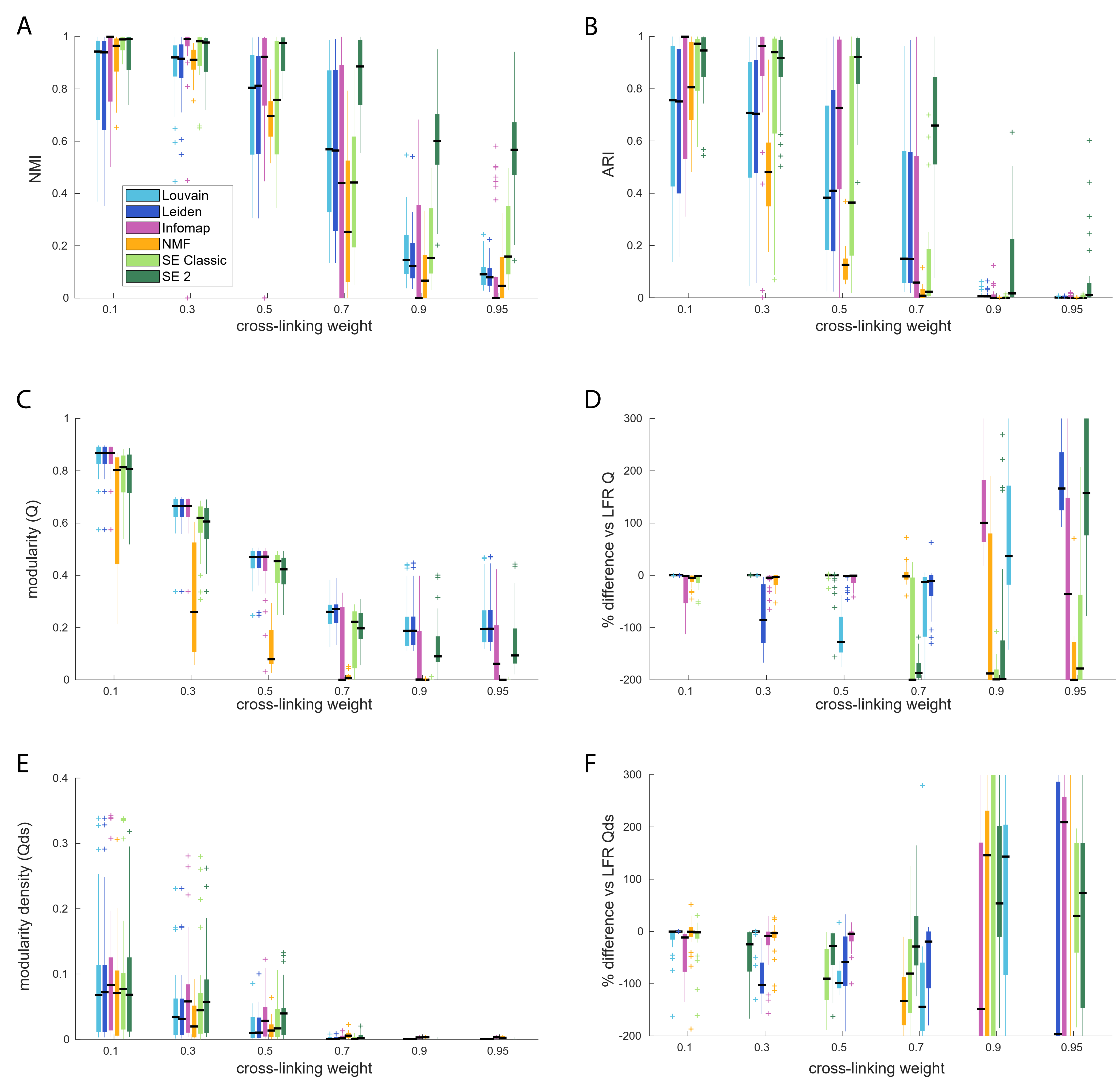


**Figure S3 (below)**


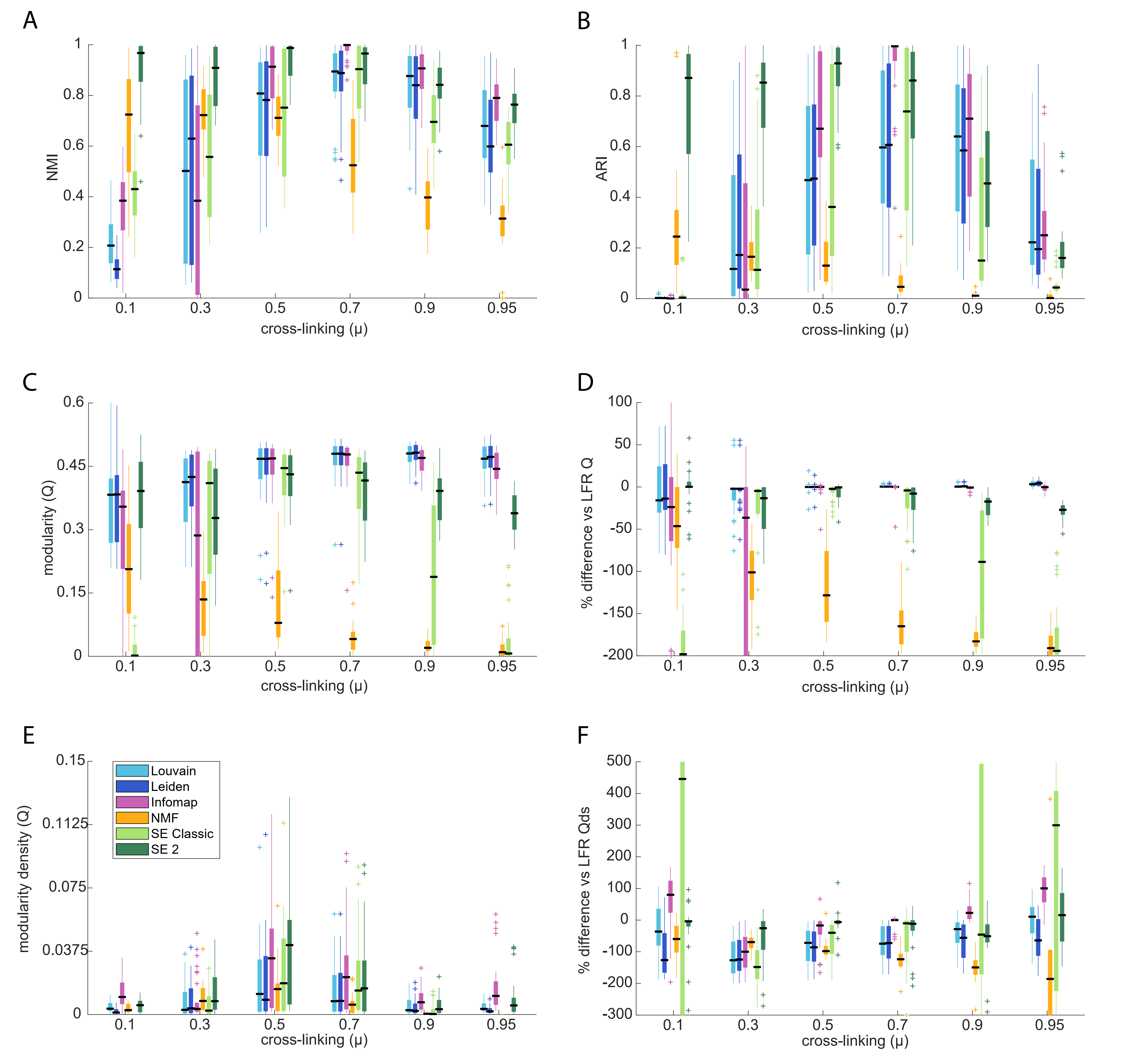


**Figure S4**


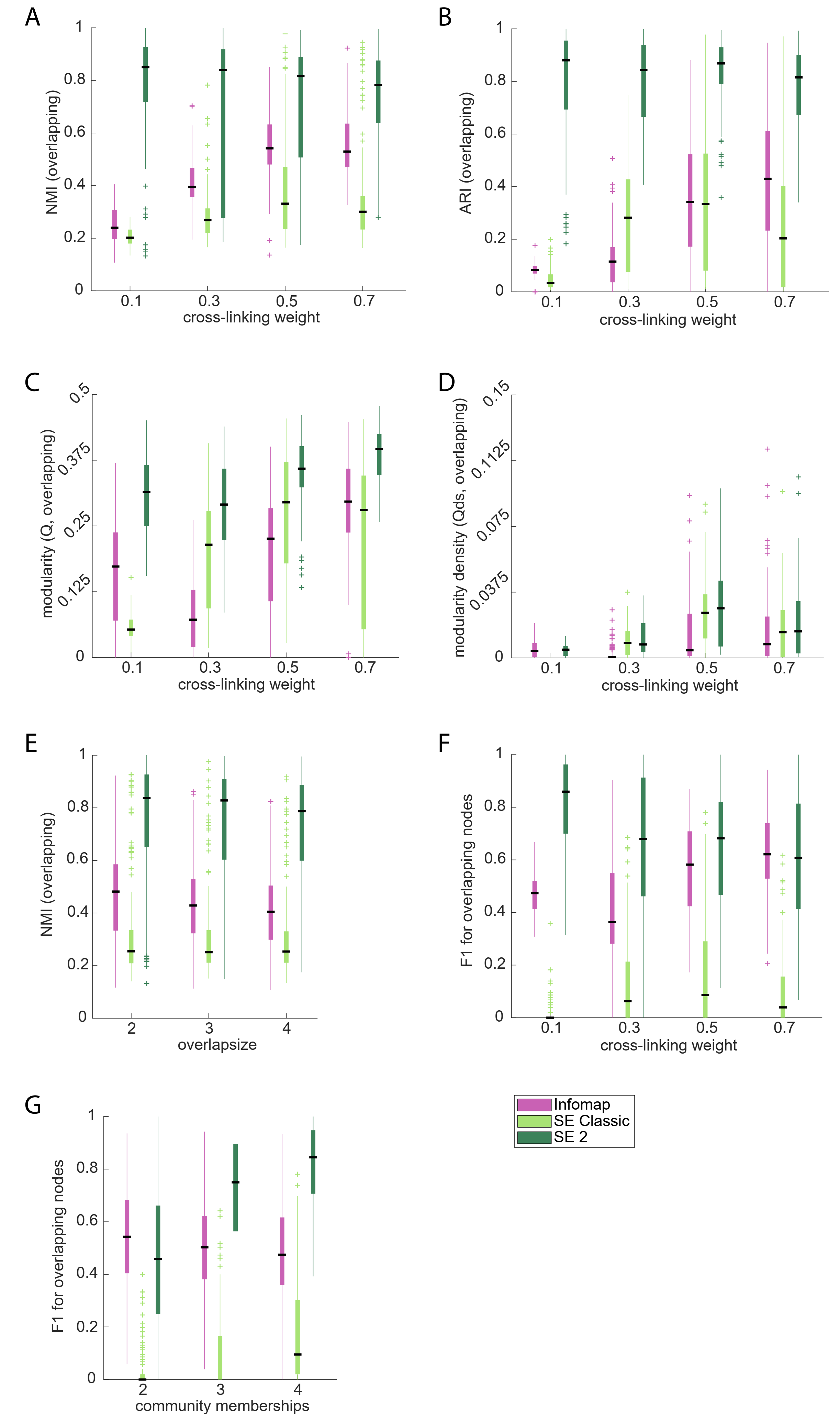


**Figure S5 (below)**


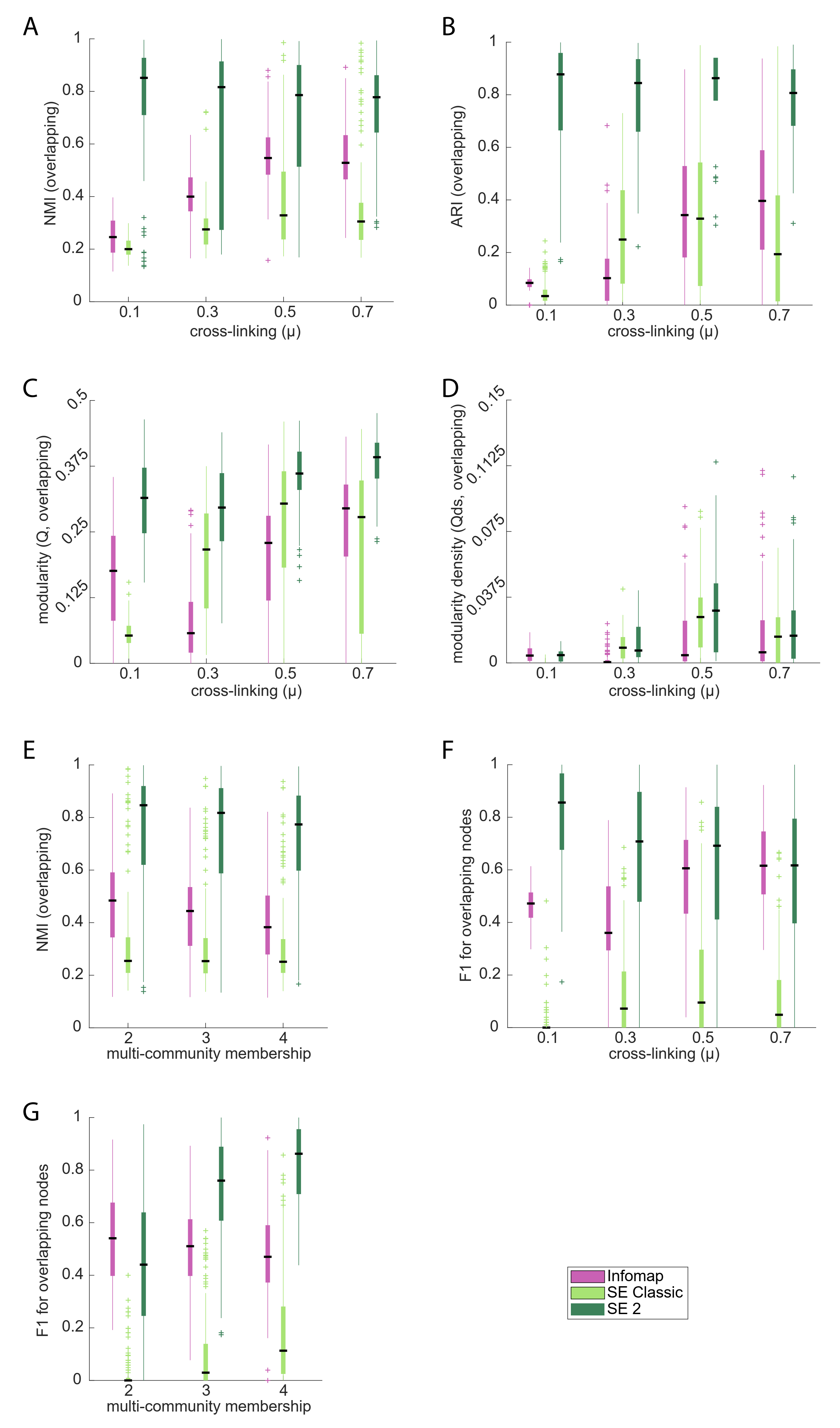


**Figure S6 (below)**


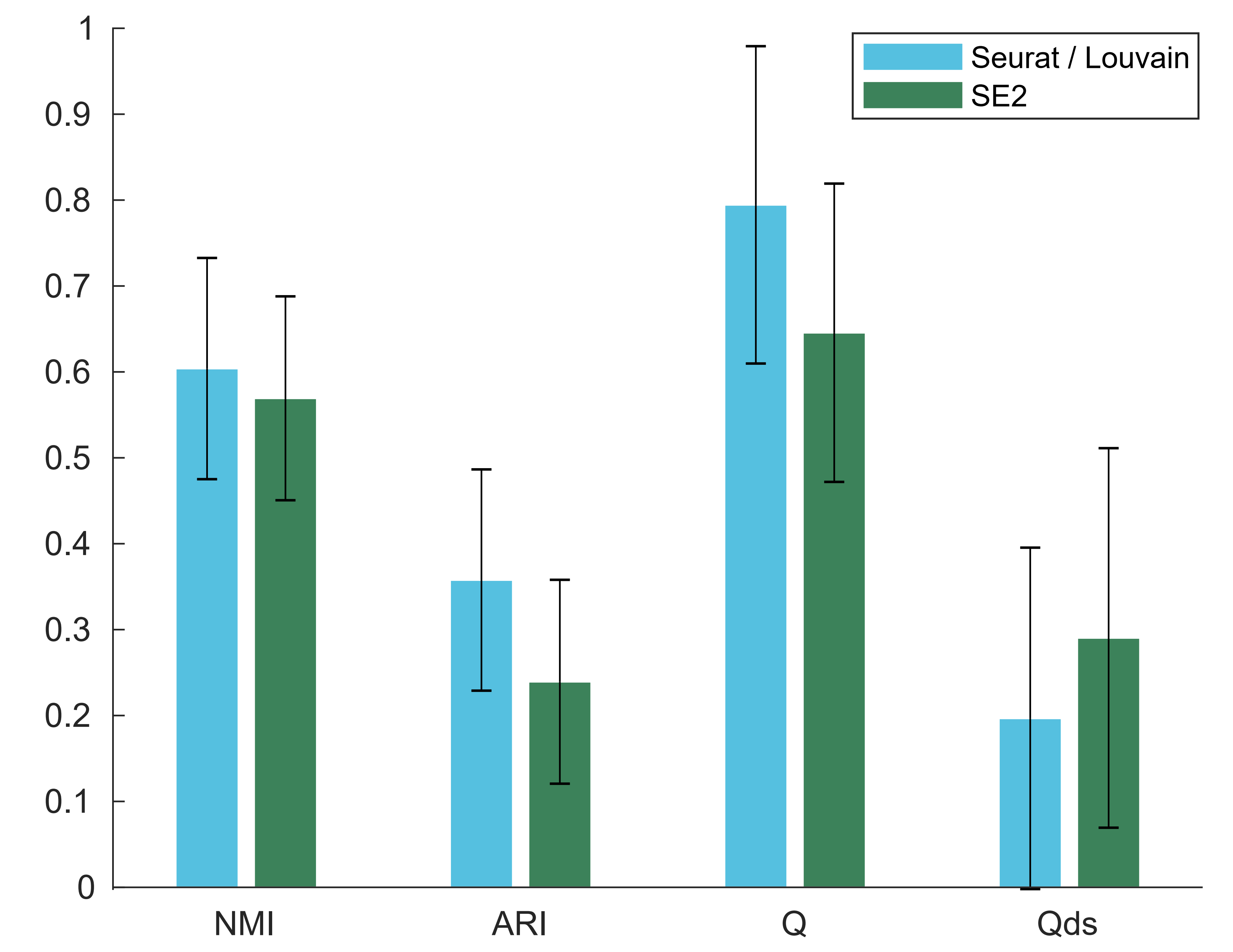


**Figure S7 (below)**


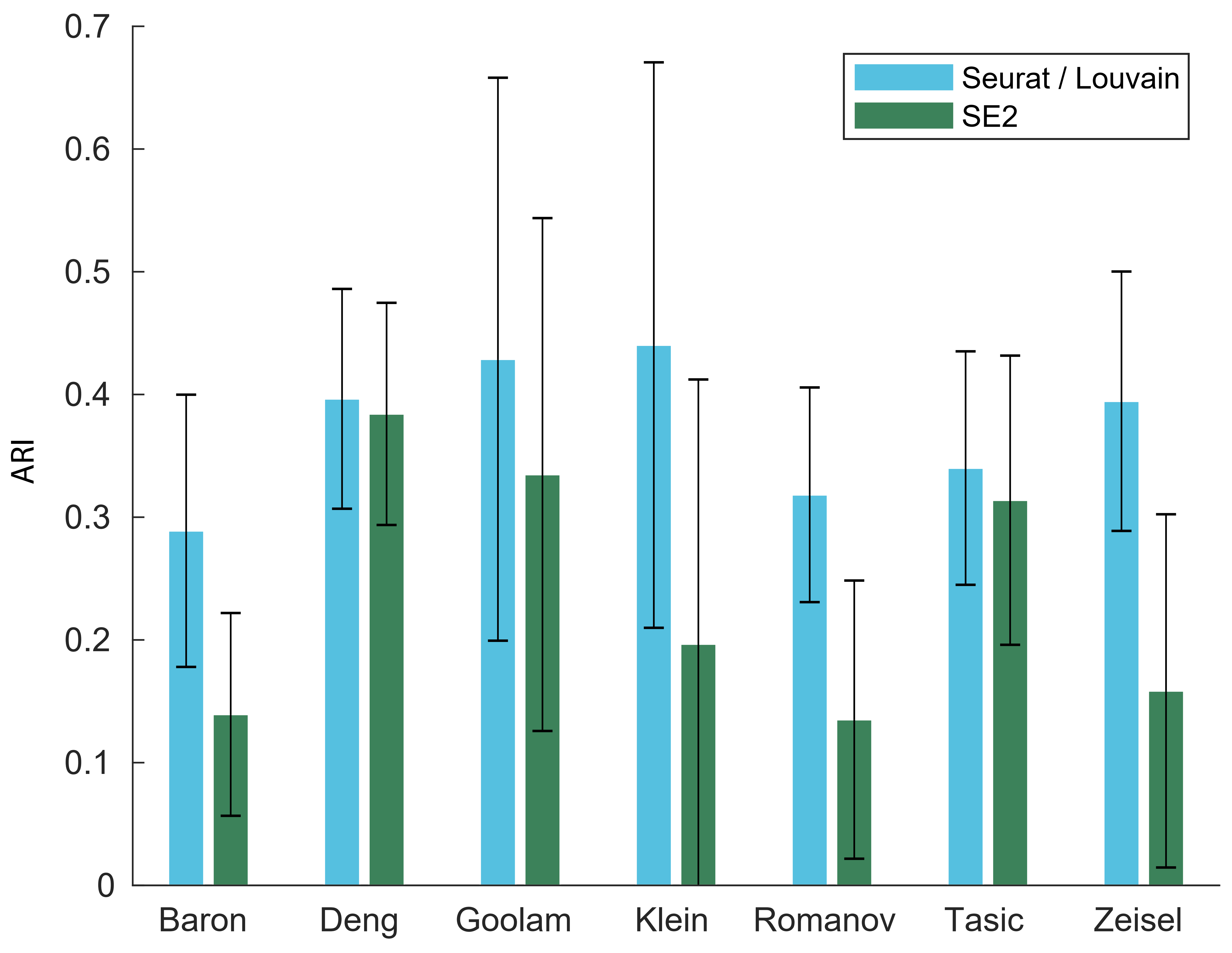


**Figure S8 (below)**


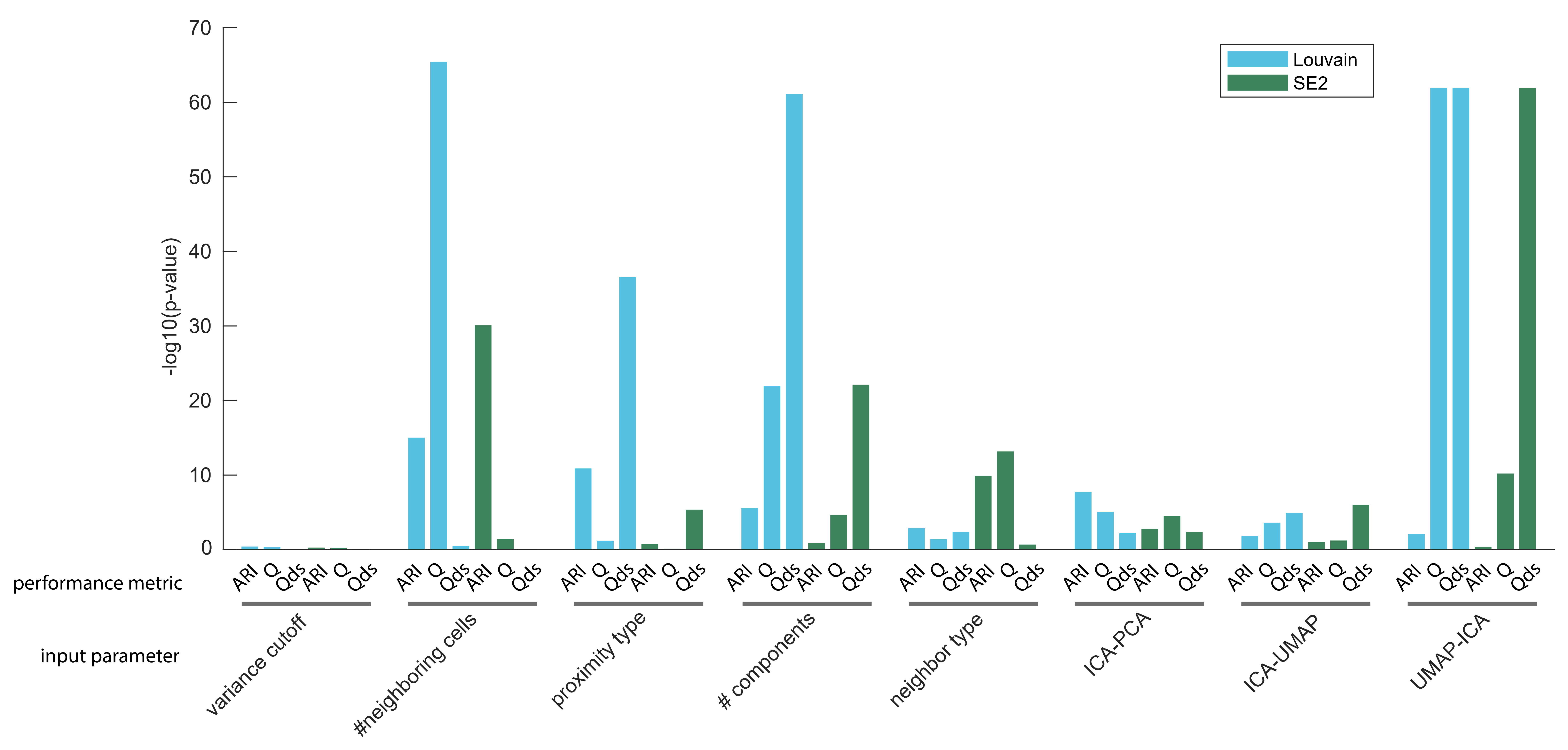


**Figure S9 (below)**


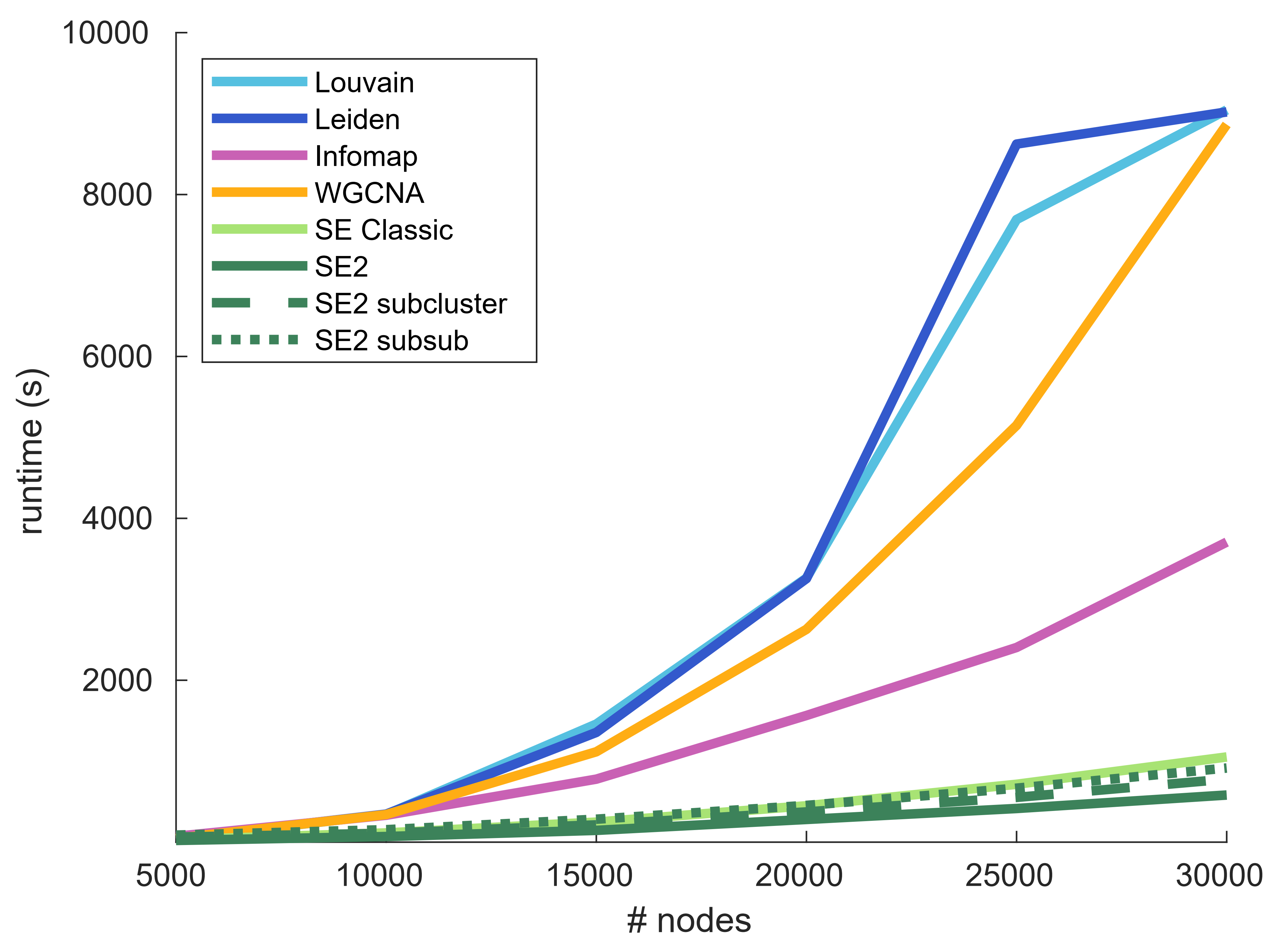


**Figure S10 (below)**


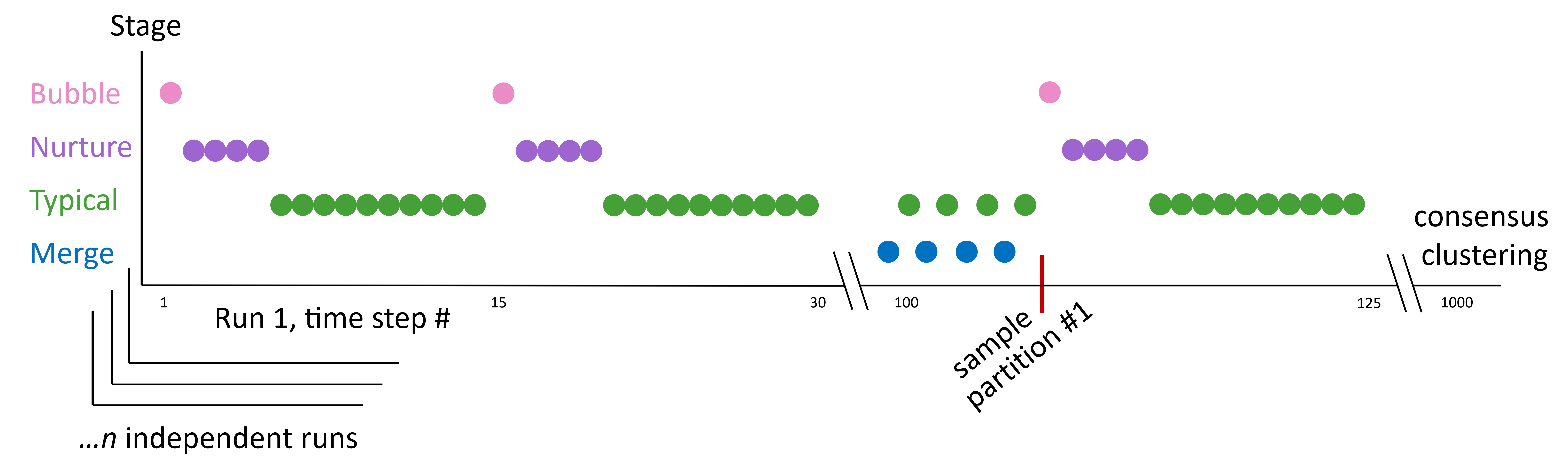

Supplement: Supplementary file 1 — Additional file 1. Supplementary text and supplementary figures. [file 13059_2023_3062_MOESM1_ESM.docx]
