# Supplementary material for: Robust, scalable, and informative clustering for diverse biological networks
Source: Genome Biol. 2023 Oct 12;24:228. doi: 10.1186/s13059-023-03062-0 (PMC10571258; doi:10.1186/s13059-023-03062-0)
Supplement: Supplementary file 5 — Additional file 5. SE2 Algorithm Pseudocode. [file 13059_2023_3062_MOESM5_ESM.pdf]

Algorithm 1 SpeakEasy2

```
function SpeakEasy2(Adjacency A)
    R ← 10                                   ▷ Set number of runs to perform
    P ← List()                               ▷ Container for possible partitions
    for i ← 1 to R do
        p ← LabelNodes(A)
        P.append(p)
    end for
    P.flatten()
    p ← SelectRepresentativePartition(P)
    return p
end function

function LabelNodes(Adjacency A)
    P ← List()
    p ← InitialLabels(A)
    merge-p ← false
    n_bubbles ← 0                            ▷ Number of times bubble stage run
    n_merged ← 0                             ▷ Number of merged community during last merge phase
    n_partitions ← 5                         ▷ Set number of partitions to find
    t ← 1
    while length(P) < n_partitions do
        M ← SelectMode(t, merge-p)
        if M is TYPICAL then
            p ← Typical(A, p)
        else if M is NURTURE then
            p ← Nurture(A, p)
        else if M is BUBBLE then
            p ← Bubble(A, p)
            n_bubbles ← n_bubbles + 1
            if n_bubbles > 2 then
                merge-p ← true               ▷ Consider merging only after a few initial bubble phases
            end if
        else if M is MERGE then
            n_bubbles ← 0
            p, n_merged ← Merge(A, p)
            if n_merged = 0 then             ▷ Only add partition if stable
                P.append(p)
                merge-p ← false              ▷ Turn off merging until partition has been bubbled
            end if
        end if
        t ← t + 1
    end while
    return P
end function

function InitialLabels(Adjacency A)
    P ← Vector(|V(A)|)                       ▷ Container for random labels
    n_labels ← ceil(|V(A)| / 100)
    for ν ∈ V(A) do
        if |{υ | (υ, ν) ∈ E(A), υ ≠ ν}| > 0 then   ▷ Check if node has incoming connections
            P[ν] ← randint(1, n_labels)
        end if
    end for
    for ν ∈ V(A) do
        l_max ← max(P)
```

if $|\{\upsilon \mid (\upsilon, \nu) \in E(A), \upsilon \neq \nu\}| = 0$ **then** ▷ Check if node has no incoming connections
    $P[\upsilon] \leftarrow l_{\max} + 1$
  end if
end for
return P
end function

function SelectMode(TimeStep t, Predicate merge-p)
  if $t <= 20$ **then**
    return TYPICAL
  end if
  if merge-p **then**
    if TimeSinceLastMerge > 1 and TimeSinceLastBubble > 3 **then**
      return MERGE
    end if
  else
    if TimeSinceLastMerge > 2 and TimeSinceLastBubble > 14 **then**
      return BUBBLE
    end if
    if TimeSinceLastMerge > 1 and TimeSinceLastBubble < 5 **then**
      return NURTURE
    end if
  end if
  return TYPICAL ▷ Default to typical mode
end function

function MostSpecificLabel(Node ν, Adjacency A, Partition P)
  $l' \leftarrow 0$ ▷ Holds current best label
  $q \leftarrow -\infty$ ▷ Holds current best label's specificity
  for $l \in \{P\}$ **do**
    $o \leftarrow \sum_{i \in A, i \neq \nu} A[i,\nu]\delta(P[i], l)$ ▷ Observed sum of neighbors labeled l
    $e \leftarrow \left(\frac{\sum_{i,j \in A, i \neq j} A[i,j]\delta(P[i],l)}{\sum_{i,j \in A, i \neq j} A[i,j]} \right) \sum_{i \in A, i \neq \nu} A[i,\nu]$ ▷ Expected sum of neighbors labeled l
    if $o - e > q$ **then**
      $l' \leftarrow l$
      $q \leftarrow o - e$
    end if
  end for
  return l', q
end function

function LowestSpecificityLabels(Adjacency A, Partition P, Threshold θ)
  $n_{\text{update}} \leftarrow \text{ceil}(\theta \times |V(A)|)$
  $Q \leftarrow$ Vector($V(A)$)
  $N \leftarrow$ Vector(n_{update})
  for $\nu \in V(A)$ **do**
    $\_, Q[\nu] \leftarrow$ MostSpecificLabel(ν, A, P)
  end for
  for $i \leftarrow 0$ **to** $i < n_{\text{update}}$ **do**
    $N[i] = Q.\text{pop}(\arg\min_{\nu}(Q))$
  end for
  return N
end function

function Typical(Adjacency A, Partition P)
  $P_{\text{updated}} \leftarrow P$
  for $\nu \in (V' \subset V(A))$ **do** ▷ Only update a subset of all nodes (90%)

```
        P_updated[ν], _ ← MostSpecificLabel(ν, A, P)
    end for
    return P_updated
end function

function Nurture(Adjacency A, Partition P)
    P_updated ← P
    N ← LowestSpecificityLabels(A, P, 0.9)                        ▷ Worst 90% of node labels
    for ν ∈ N do                                                  ▷ Only update poorly labeled nodes
        P_updated[ν], _ ← MostSpecificLabel(ν, A, P)
    end for
    return P_updated
end function

function Bubble(Adjacency A, Partition P)
    L ← {{ν | ν ∈ V(A), P[ν] = l} | l ∈ {P}}                      ▷ Sets of nodes in each label
    P_updated ← P
    N ← LowestSpecificityLabels(A, P, 0.9)                        ▷ Worst 90% of node labels
    ñ ← median({|l| | l ∈ L})                                     ▷ Median community size
    for l ∈ L do                                                  ▷ Set of nodes in label
        l_max ← max(P_updated)
        if |l| > 4 then                                           ▷ Don't burst small communities
            for ν ∈ (l ∩ N) do                                    ▷ Low quality nodes in the community
                P_updated[ν] ← randint(1, |(l∩N)|/ñ) + l_max      ▷ Randomly assign to new communities[1][2]
            end for
        end if
    end for
    return P_updated
end function

function Merge(Adjacency A, Partition P)
    L ← Vector(|P|)                    ▷ Container for label that sends most crosstalk to i_th label
    C ← Vector(|P|)                    ▷ Container for amount of crosstalk sent by label in L
    P_updated ← P
    n_merged ← 0
    for l ∈ {P} do
        L[l], C[l] ← MaxCrossTalk(A, P, l)
    end for
    c̄ ← mean(C)
    for l ∈ {P} do
        if C[l] > c̄, L[l] ∈ {P_updated} then        ▷ Ensure L[l] hasn't already been merged into l
            for ν ∈ V(A) do
                if P[ν] = l then
                    P_updated[l] ← L[l]
                end if
            end for
            n_merged ← n_merged + 1
        end if
    end for
    return P_updated, n_merged
end function
                                  ▷ How much nodes in one label heard from nodes in another label
function MaxCrosstalk(Adjacency A, Partition P, Label l)
    l′ ← 0                                                        ▷ Holds current highest crosstalk label
```

[1] New communities should be approximately the size of the median community size.

[2] New communities are split from old communities; nodes can't end up in a community with a node that was previously in a different community.

$c \leftarrow -\infty$ ▷ Holds current highest amount of crosstalk
for $l_i \in \{P\}, l_i \neq l$ **do**
    $o \leftarrow \sum_{i,j \in A, i \neq j} A[i,j]\delta(P[i], l_i)\delta(P[j], l)$ ▷ Sum of nodes in l_i that talk to a node in l
    $e \leftarrow \left(\frac{\sum_{i,j \in A, P[i] \neq P[j]} A[i,j]\delta(P[i], l_i)}{\sum_{i,j \in A, P[i] \neq P[j]} A[i,j]} \right) \sum_{i,j \in A, P[i] \neq P[j]} A[i,j]\delta(P[j], l)$ ▷ Expected talk from l_i into l
    if $o - e > q$ **then**
        $l' \leftarrow l$
        $q \leftarrow o - e$
    end if
end for
return l', c
end function

function SelectRepresentativePartition(Partitions P)
    $p_{\text{best}} \leftarrow 0$ ▷ Most representative partition
    $q_{\text{best}} \leftarrow 0$ ▷ Sum of most representative partition's NMIs
    for $p_i \in P$ **do**
        $q \leftarrow \sum_{p_j \in P, p_j \neq p_i} \text{NMI}(p_i, p_j)$
        if $q > q_{\text{best}}$ **then**
            $p_{\text{best}} \leftarrow p_i$
            $q_{\text{best}} \leftarrow q$
        end if
    end for
    return p_{best}
end function
