# Supplementary figures and images for: Robust, scalable, and informative clustering for diverse biological networks
Source: Genome Biol. 2023 Oct 12;24:228. doi: 10.1186/s13059-023-03062-0 (PMC10571258; doi:10.1186/s13059-023-03062-0)

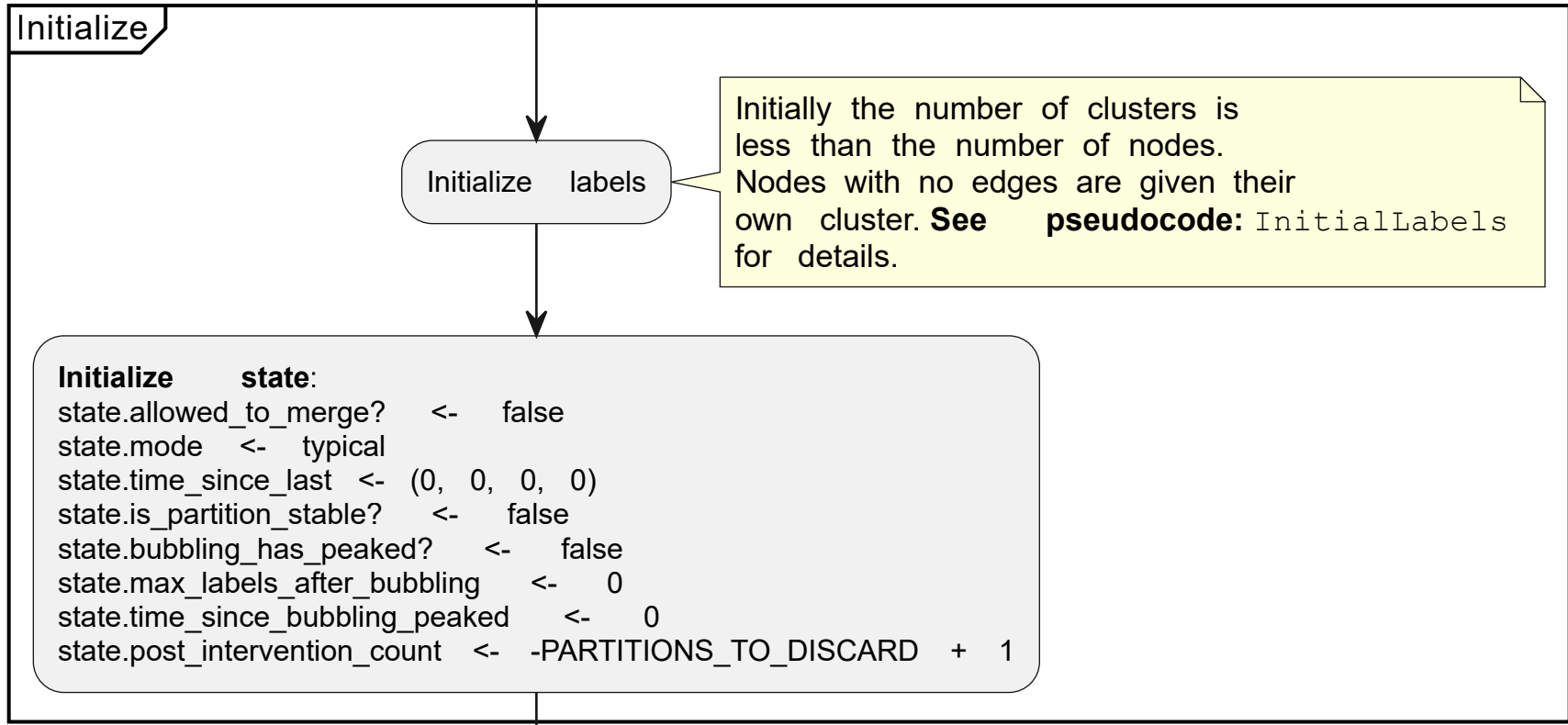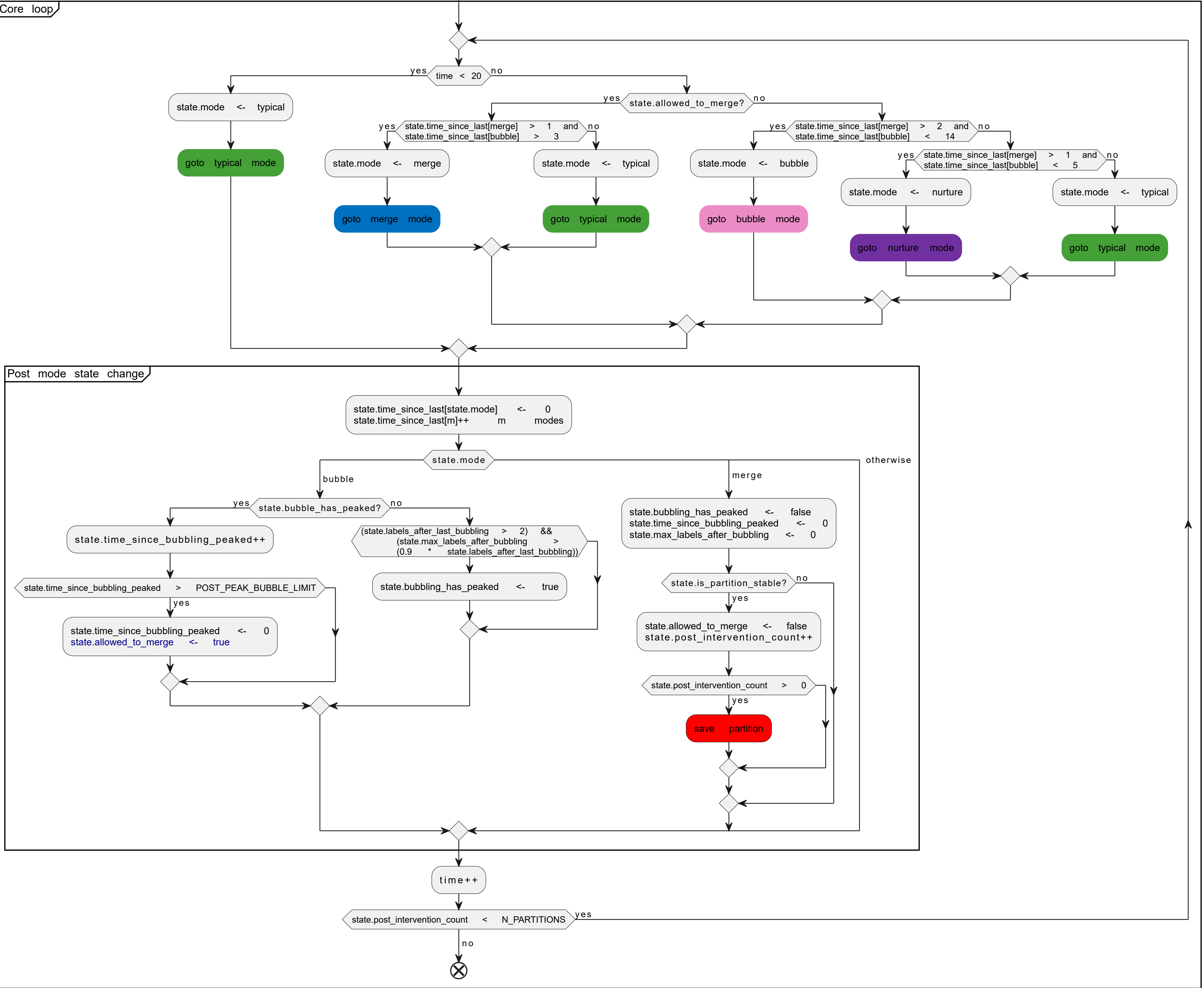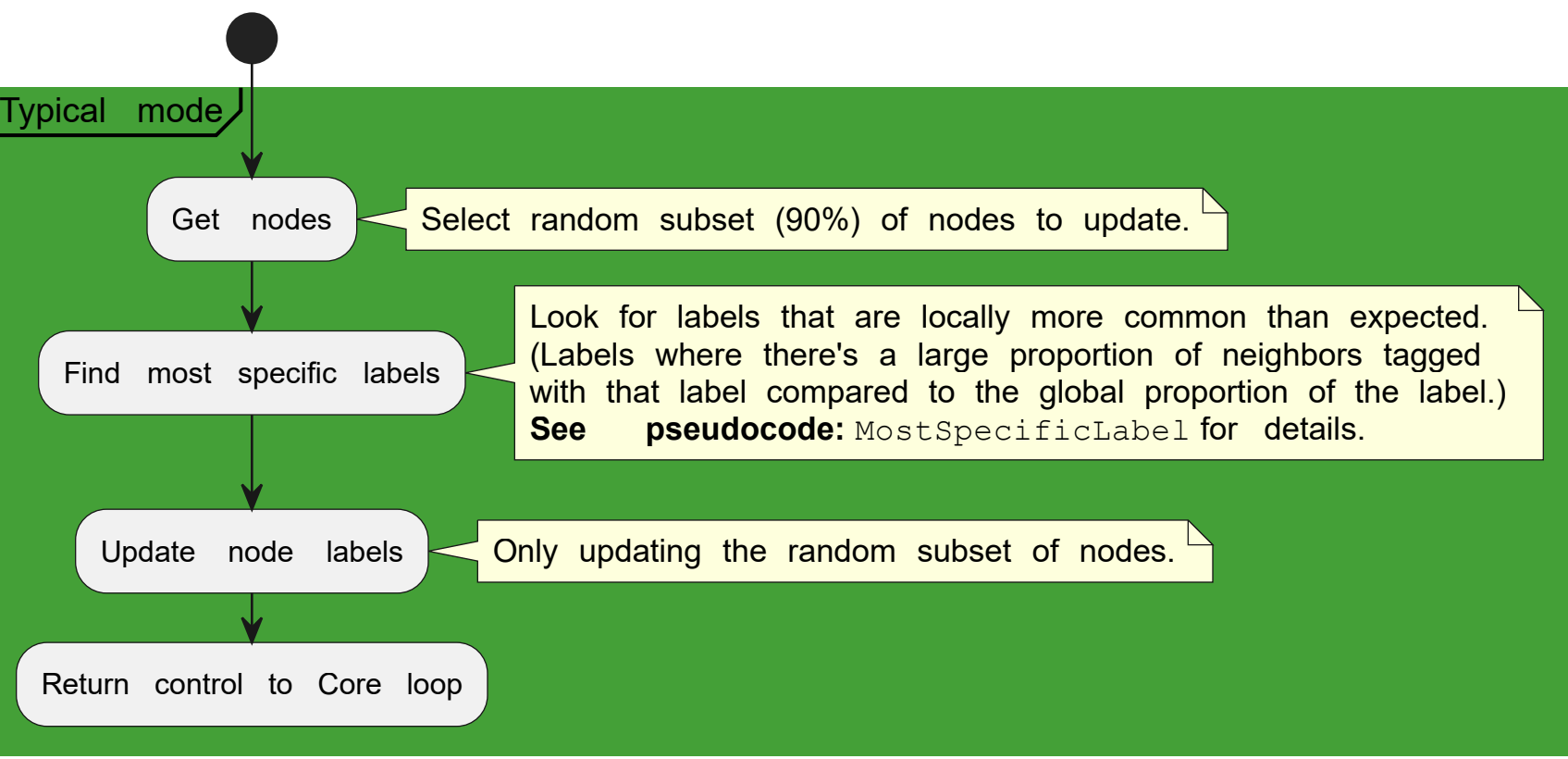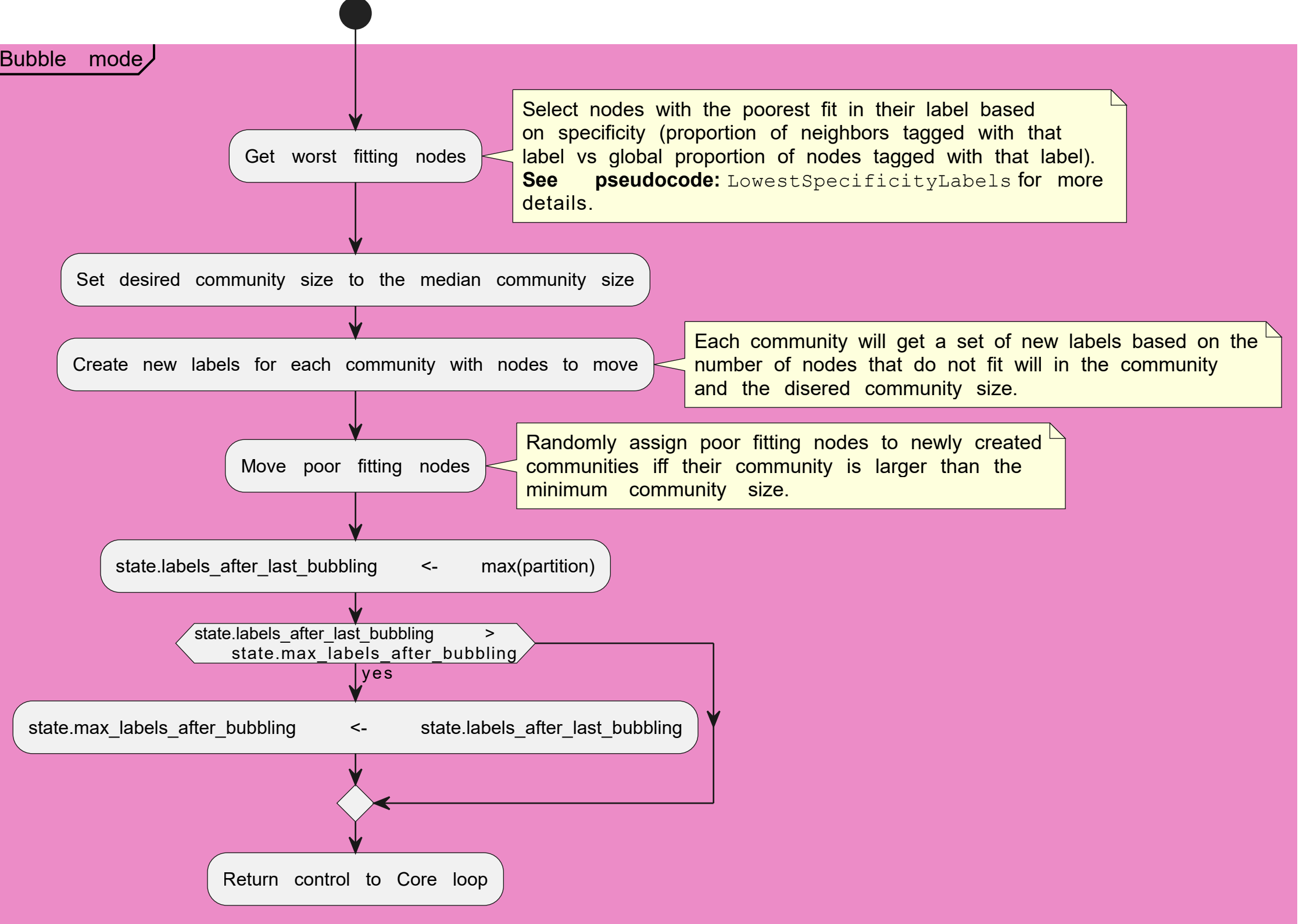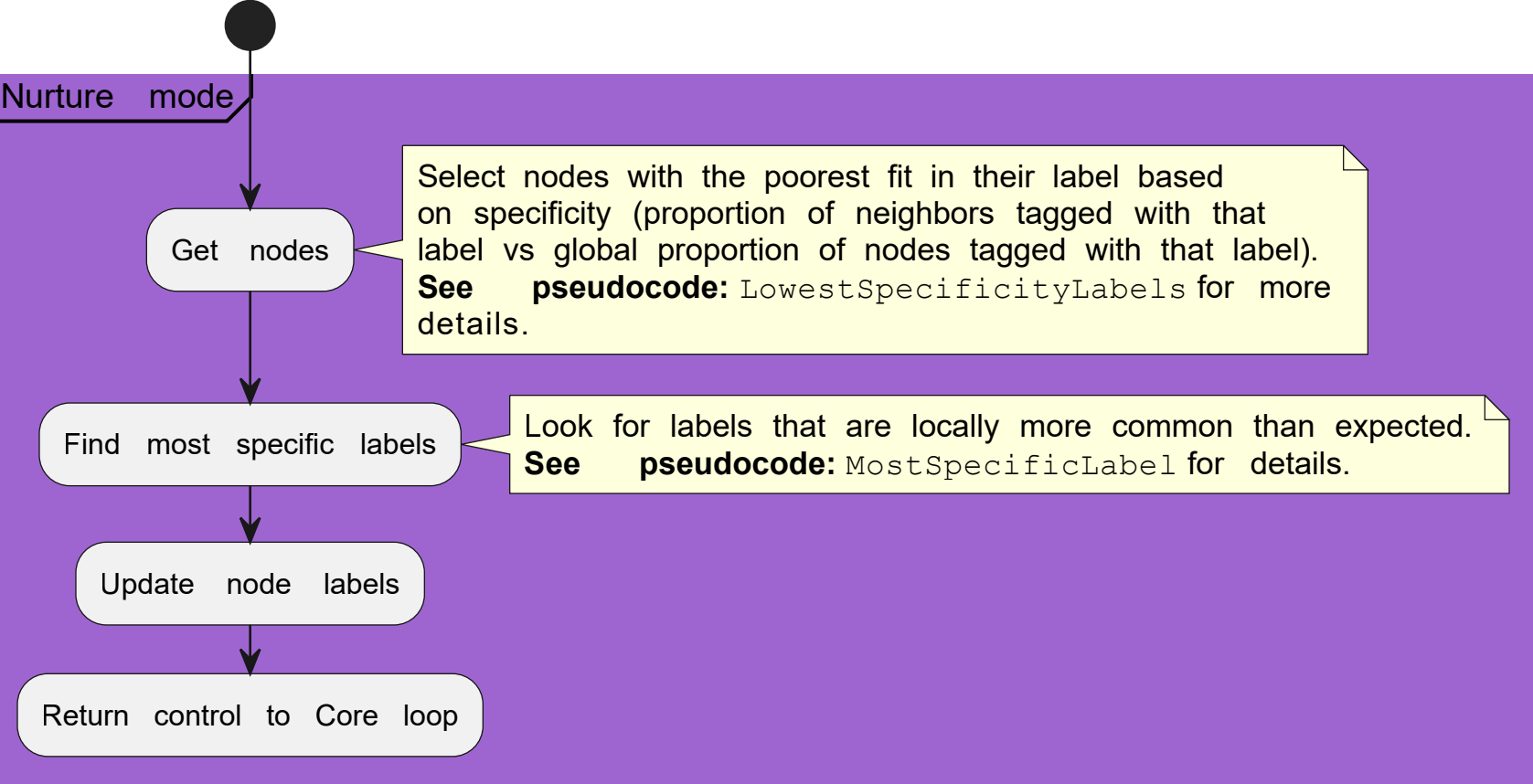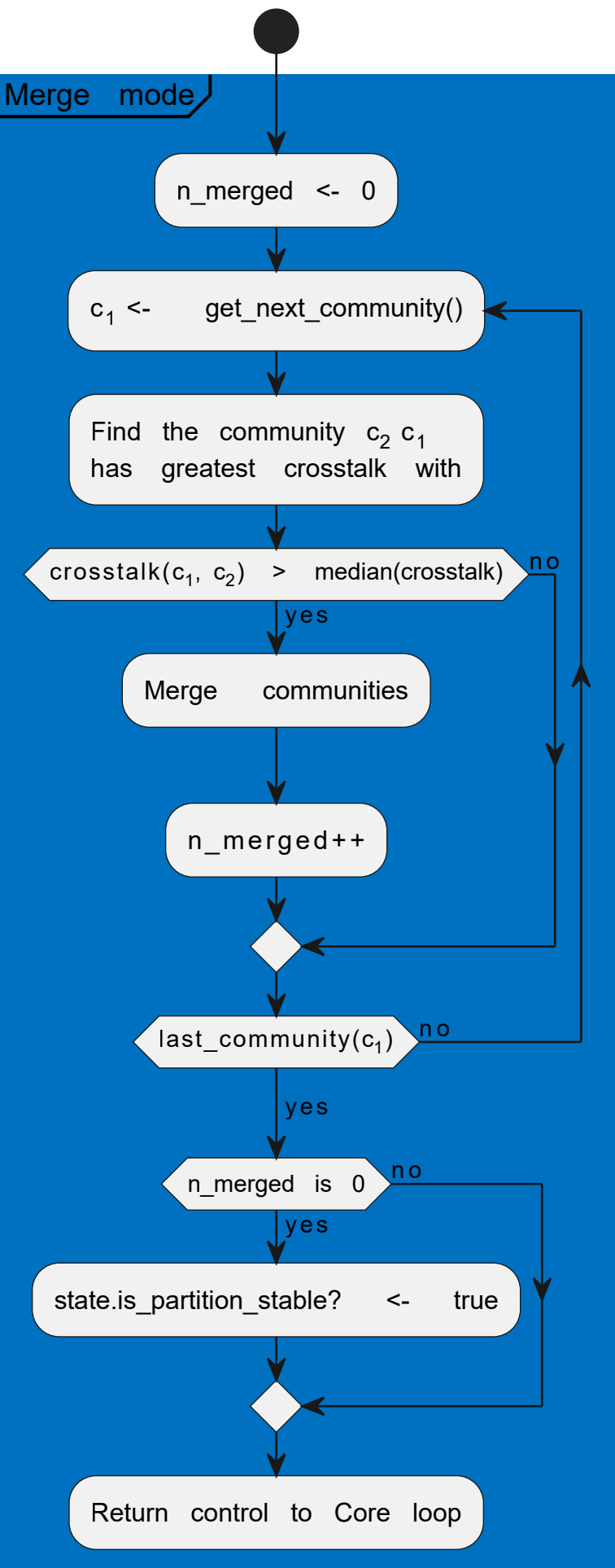

Supplement: Supplementary file 6 — Additional file 6. Global flowchart of all SE2 operations. [file 13059_2023_3062_MOESM6_ESM.pdf]
