# Supplementary material for: Robust, scalable, and informative clustering for diverse biological networks
Source: Genome Biol. 2023 Oct 12;24:228. doi: 10.1186/s13059-023-03062-0 (PMC10571258; doi:10.1186/s13059-023-03062-0)
Supplement: Supplementary file 7 — Additional file 7. Review history. [file 13059_2023_3062_MOESM7_ESM.docx]

Review History

**First round of review**

**Reviewer 1**

**Were you able to assess all statistics in the manuscript, including the appropriateness of statistical tests used?** Yes, and I have assessed the statistics in my report.

**Comments to author:**

The article introduces SE2, which is an improved version of a previously developed clustering method named SpeakEasy (SE). SE2 is a community detection algorithm based on labeling propagation between joint communities. This algorithm can detect non-overlapping and overlapping communities. Using this algorithm, the multi-community nodes can be identified as well. The authors have provided an extensive set of experiments to show the superiority of SE2 (SpeakEasy2) over other clustering approaches. These experiments are performed on multiple sets of networks, including overlapping and non- overlapping clustering on the LFR benchmark set of networks, bulk gene expression data, single-cell datasets, and proteins interactions networks. Also, the algorithm is tested on extremely large networks. This demonstrates the applicability of SE2 in clustering large networks where many other methods would fail. The results are discussed thoroughly for each set of experiments. This extensive set of experiments would avoid skewness of the algorithm towards a specific parameter or set of networks. However, there are some major issues that need to be addressed carefully. The comments are as follows:

1. The authors claim the SE2 improves the run-time complexity. However, no analysis and comparison were done to prove this claim. Authors should provide the running time for each experiment that they have done.

2. SE2 should be compared with the latest version of Louvain named Leiden. Leiden has been showed significant improvement over Louvain in both time and performance complexity.

3. There is no clear connection between the reported results and modifications to the SE algorithm. It would be helpful if authors could relate the improvements in accuracy to a specific part of their modification of the classic SE algorithm.

4. The authors should provide a method figure that describe their method at the high-level. In addition, the authors can provide the pseudo-code of SE2 like the classical SE paper in supplementary. The altered parts of the SE algorithm in the new version (SE2) can be highlighted in a different color. It will help readers to follow the method easier.

5. In bulk gene expression experiments, authors can test their algorithm using more datasets. At the moment, the algorithm is tested on only one dataset. This will avoid bias in the reported results.

6. Besides the description in the main text, the analysis results of gene expression are better to be prepared as figures like the reported results on the LFR networks set.

7. In the single cell experiments, besides text, the results per each dataset and averaged results should be presented as figures/tables (maybe in supplementary). The results are explained in a long text which makes it hard to follow and evaluate.

8. In the zoomed area of Figure 7, the agreement between ground truth and Louvain can be shown similarly to SE2 (circles with different colors). From the zoomed region, there is no difference between the partitioned proteins network by Louvain and SE2. Showing another aspect of the clustered network can be more intuitive in terms of the superiority of SE2 to Louvain.

9. The results of gene interaction networks would be more understandable if they are plotted. These plots can be shown in the supplementary material.

10. The results on large networks can be shown in a detailed table to follow in what scale of large networks each algorithm stops. As stated in another comment, it is highly recommended that the run time is also included in the reported results.

11. In all experiments (all sets of networks in different sections such as single cell, bulk gene expression, etc.), the classic SE should be participated in the comparisons. So, the differences between the original SE and the proposed SE2 would be more intuitive.

12. The mentioned tables (Table 1 and Table 2) are not included in the manuscript. It could be either happened because of an error in the submission process or a corrupted manuscript. Therefore, I could not evaluate the authors' results presented in these tables. The authors should make sure all their materials are submitted correctly.

13. The manuscript needs to be revised carefully. There are some grammatical and typos that need to be corrected. For example, on page 25, line number 601, "13% (under the CYC2008 (Pu et al. 2009) "ground truth" definitions of protein complexes)," does not make any sense. Also, on page 6 lines 99-101 are repetitive.

14. The authors should check supplementary note to fix grammatical and typos.

**Reviewer 2**

**Were you able to assess all statistics in the manuscript, including the appropriateness of statistical tests used?** Yes, and I have assessed the statistics in my report.

**Comments to author:**

This paper contains a nice algorithmic contribution, that is probably publishable, with some extensive benchmarking but the framing of that contribution in the introduction is weakened by some over-hype, as well as lacking some mathematical crispness that would strengthen the authors' points. It appears that they are trying to make some sort of universal claim of superiority (also see the discussion section), while at the same time debunking notions that any clustering method can be universally superior.

I would like the authors to reframe their introduction by talking about the specific clustering method they introduce up front, Speakeasy2. They then can argue that its principal contribution is that it is able to produce sensible clusters out of the box, which are stable (define better what you mean by stability mathematically, see below), without requiring massive amounts of parameters tuning as many other methods do. They then should say they test their algorithm against competitors on a wide variety of biological and nonbiological datasets.

Instead of a grandiose section called "Summary and critiques of classes of clustering methods," which seems to imply that no one knows basic issues which are well known in the field, I would suggest that the authors recast this section to categorize the classes of competitor methods they test Speakeasy2 against. Then they can remark on the strengths and weaknesses of all the methods in each class in general. They should not forget it make is clear how Speakeasy2 differs from the original Speakeasy.

The next section meanders too much. It would be better to list all the evaluation tests of non-overlapping clusters in one place. 1) Non-overlapping clusters in synthetically-generated LFR networks 2) Tests of modularity and functional coherence on real gene expression data. 3) Tests on single cell data 4) The non-overlapping tests of modularity in the PPI networks. The exact parameter settings used to construct the networks are hard to gather because they appear all over the narrative.

I suggest moving the evaluation on overlapping clusters in the synthetically-generated LFR networks, the gene expression datasets, and the PPI complexes to a separate section, since most methods do not generalize to this setting and the results are not as thorough.

I suggest moving the evaluations of the non-biological datasets to the supplementary material.

Specific comments on the introduction:

Line 30: The Louvain algorithm is not guaranteed to produce clusters with optimum modularity because it's a heuristic that can get stuck in local minima. Suggest replacing "to produce partitions" with "a heuristic that aims to produce partitions…"

Line 31: Insert the word non-overlapping: (a collection of non-overlapping clusters)

Line 39: Rephrase. As you say below, for many datasets the answer isn't unique

Lines 49, 70, and 75: "limits on reproducibility" and later "stability" and "limits of reliability" Can you define these terms more crisply and precisely?

Line 777: the authors claim that for computational reasons they cap the number of labels in the network at any given time. Is this for all the networks, or only the large ones? Is this fully described in the methods section (lines 822-871)?

Line 879: The authors return the partition they obtain with the best NMI. Do their clusters have better NMI than alternative methods that are trying instead to optimize modularity?

Finally, the main claim that makes Speakeasy2 so interesting, is this idea that it correctly and automatically, in a fashion similar to Louvain, will determine the "correct" number of clusters rather than require this as a separate input parameter. The authors should construct a synthetic dataset (different from the LFR benchmarks) that builds in clustering at several scales artificially. It would be super interesting to learn if Speakeasy2 always found the coarser or always found the finer partition, or if it varied by dataset, or …. I would encourage the authors to do this type of experiment to strengthen the conclusions of the paper.

**Authors Response**

**Point-by-point responses to the reviewers’ comments:**

Reviewer reports:

Reviewer #1: The article introduces SE2, which is an improved version of a previously developed clustering method named SpeakEasy (SE). SE2 is a community detection algorithm based on labeling propagation between joint communities. This algorithm can detect non-overlapping and overlapping communities. Using this algorithm, the multi-community nodes can be identified as well. The authors have provided an extensive set of experiments to show the superiority of SE2 (SpeakEasy2) over other clustering approaches. These experiments are performed on multiple sets of networks, including overlapping and non- overlapping clustering on the LFR benchmark set of networks, bulk gene expression data, single-cell datasets, and proteins interactions networks. Also, the algorithm is tested on extremely large networks. This demonstrates the applicability of SE2 in clustering large networks where many other methods would fail. The results are discussed thoroughly for each set of experiments. This extensive set of experiments would avoid skewness of the algorithm towards a specific parameter or set of networks. However, there are some major issues that need to be addressed carefully. The comments are as follows:

1. The authors claim the SE2 improves the run-time complexity. However, no analysis and comparison were done to prove this claim. Authors should provide the running time for each experiment that they have done.

**Response:** Thank you for noting this - we have updated the paper and supplement to include a formal complexity analysis as well as practical runtime results on a wide range of networks (Table 2 and Figure S6).

2. SE2 should be compared with the latest version of Louvain named Leiden. Leiden has been showed significant improvement over Louvain in both time and performance complexity.

**Response:** Based on your suggestion, we tested Leiden on all of the LFR discrete benchmarks and added those to the graphs and text. In brief, performance is highly correlated with Louvain on LFR – essentially the same NMI/ARI, slightly higher modularity and slightly lower modularity density.

3. There is no clear connection between the reported results and modifications to the SE algorithm. It would be helpful if authors could relate the improvements in accuracy to a specific part of their modification of the classic SE algorithm.

**Response:** At your suggestion, we isolated each of contributions of the changes in SE2 to performance and included a description of this in the supplement. The punchline is that they all contribute significantly, but the greatest is the cluster-splitting stage.

4. The authors should provide a method figure that describe their method at the high-level.

**Response:** We agree this sort of intuitive description is important. We have updated it in figure 1 to provide this sort of intuitive grasp of the method and added comment to the figure captions to indicate where it diverges from the original algorithm.

In addition, the authors can provide the pseudo-code of SE2 like the classical SE paper in supplementary. The altered parts of the SE algorithm in the new version (SE2) can be highlighted in a different color. It will help readers to follow the method easier.

**Response:** We added a new section in the supplement with the full algorithm in pseudo-code indicating where it is different from the original algorithm.

5. In bulk gene expression experiments, authors can test their algorithm using more datasets. At the moment, the algorithm is tested on only one dataset. This will avoid bias in the reported results.

**Response:** We added the next two largest RNAseq datasets we could find (generated by other institutions) and added their results to the paper. We also devised additional tests of cluster quality for these smaller data, with results showing that SE2 provides clusters twice as stable as WGCNA, while those clusters simultaneously have higher modularity.

6. Besides the description in the main text, the analysis results of gene expression are better to be prepared as figures like the reported results on the LFR networks set.

**Response**: See #9

7. In the single cell experiments, besides text, the results per each dataset and averaged results should be presented as figures/tables (maybe in supplementary). The results are explained in a long text which makes it hard to follow and evaluate.

**Response:** On lines 499-510 we describe the benefits of showing the similarities of these datasets in a network-style display, so that readers can see how performance varies by preprocessing, metric and. In contrast, if readers just glance at a barplot to see method x is better (and in this case there is no such clear winner), that defeats part of the point of the diverse testing, because results are highly variable based on input preprocessing and there’s not an overall winner. We understand the point of trying to compress the results as we do with LFR and then mentioning when performance interacts with dataset parameters, but reason to emphasize diversity here is because there are actually different datasets and there are some non-linear relationships of parameters to response, which are most easily shown graphically.

8. In the zoomed area of Figure 7, the agreement between ground truth and Louvain can be shown similarly to SE2 (circles with different colors). From the zoomed region, there is no difference between the partitioned proteins network by Louvain and SE2. Showing another aspect of the clustered network can be more intuitive in terms of the superiority of SE2 to Louvain.

**Response:** Image revised as you suggest. Please let us know if you prefer the end result to the original. Reason for asking is we wonder if it makes the large clusters of Louvain less obvious in the zoomed-out view. Let us know which version you prefer and we can go with that.

9. The results of gene interaction networks would be more understandable if they are plotted. These plots can be shown in the supplementary material.

**Response:** There are indeed many results, each of which generally has different variables and conditions. Therefore, we don’t think generating 20 bar plots (two bars each) is going to be helpful, because the reader just has to go back to the text to figure out which each is why it’s important. More broadly you can see we’re very open to suggestions, so hope this is acceptable for the reason stated.

10. The results on large networks can be shown in a detailed table to follow in what scale of large networks each algorithm stops. As stated in another comment, it is highly recommended that the run time is also included in the reported results.

**Response:** At your suggestion we have added runtime results to that table. The other information you request is included in the table already, so we asked the editors to make sure it transmits properly.

11. In all experiments (all sets of networks in different sections such as single cell, bulk gene expression, etc.), the classic SE should be participated in the comparisons. So, the differences between the original SE and the proposed SE2 would be more intuitive.

**Response:** For single cell, there is really only one method used (Louvain) – no one has ever tried used original SE for it (including us). Since you already mentioned results on that were complex, we didn’t think it was worth putting in another method that no one is ever going to use in that application, especially now that SE2 is available.

12. The mentioned tables (Table 1 and Table 2) are not included in the manuscript. It could be either happened because of an error in the submission process or a corrupted manuscript. Therefore, I could not evaluate the authors' results presented in these tables. The authors should make sure all their materials are submitted correctly.

**Response**: On our end, we can see those tables in the “submitted” version, so we’re not sure what happened, but we will ask the editors about this.

13. The manuscript needs to be revised carefully. There are some grammatical and typos that need to be corrected. For example, on page 25, line number 601, "13% (under the CYC2008 (Pu et al. 2009) "ground truth" definitions of protein complexes)," does not make any sense. Also, on page 6 lines 99-101 are repetitive.

**Response:** Thank you for these notes – we have fixed those issues.

14. The authors should check supplementary note to fix grammatical and typos.

**Response:** All typos are corrected.


Reviewer #2: This paper contains a nice algorithmic contribution, that is probably publishable, with some extensive benchmarking but the framing of that contribution in the introduction is weakened by some over-hype, as well as lacking some mathematical crispness that would strengthen the authors' points. It appears that they are trying to make some sort of universal claim of superiority (also see the discussion section), while at the same time debunking notions that any clustering method can be universally superior.

**Response:** We appreciate your careful reading and that our changes in response to your questions have prompted us to improve this research. We have designed further tests that we think answer your main technical question detail. For comment above, we have modified the text because indeed we strive to provide an algorithm which is useful in many biological contexts. As you point out, there are mathematical limits on how successful that mission can be, so we are motivated to perform exhaustive testing to find where it fails. We point this out more clearly in the introduction.

I would like the authors to reframe their introduction by talking about the specific clustering method they introduce up front, Speakeasy2. They then can argue that its principal contribution is that it is able to produce sensible clusters out of the box, which are stable (define better what you mean by stability mathematically, see below), without requiring massive amounts of parameters tuning as many other methods do. They then should say they test their algorithm against competitors on a wide variety of biological and nonbiological datasets. Instead of a grandiose section called "Summary and critiques of classes of clustering methods," which seems to imply that no one knows basic issues which are well known in the field, I would suggest that the authors recast this section to categorize the classes of competitor methods they test Speakeasy2 against. Then they can remark on the strengths and weaknesses of all the methods in each class in general. They should not forget it make is clear how Speakeasy2 differs from the original Speakeasy.

**Response:** Okay, reorganized as you suggest. With regard to our inclusion of notes on the limits of clustering, this is directly motived by experience listening to many biology presentations wherein 95+% of those biologists are clearly unaware of these foundational issues. For most researchers in that audience, we find that every cluster is perceived to be intrinsically robust, and are inevitably surprised to hear any of these limitations. They also have no idea what their major options are for clustering, and no knowledge of clustering beyond whatever method they’re currently using. This is the reality we’re are trying to address with our comments on the limitations of clustering in a journal that most biologist would read. We have zero intention of being “grandiose”, so if you see specific evidence of that beyond the topic of discussion itself (talking about clustering limitations doesn’t seem intrinsically grandiose), please point it out and we’ll fix it. In our efforts to reach biologists, we certainly don’t want to offend any one! Trying to be clearer about our intended audience for these comments, we have elevated this issue into sentences 1&2 of the intro.

The next section meanders too much. It would be better to list all the evaluation tests of non-overlapping clusters in one place. 1) Non-overlapping clusters in synthetically-generated LFR networks 2) Tests of modularity and functional coherence on real gene expression data. 3) Tests on single cell data 4) The non-overlapping tests of modularity in the PPI networks. The exact parameter settings used to construct the networks are hard to gather because they appear all over the narrative. I suggest moving the evaluation on overlapping clusters in the synthetically-generated LFR networks, the gene expression datasets, and the PPI complexes to a separate section, since most methods do not generalize to this setting and the results are not as thorough.

**Response:** We have cut results from most sections to reduce length. I actually organized this section as you suggest, but then multiple biologists on our team said they thought it did *not* make sense and asked for the current order, because the biological applications are ordered by biophysical scale (genes then proteins then cells etc). They wanted all the synthetic results in one place because they are mentally grouping result by application type, rather than the underlying data structure. Since our audience is mainly biologists, I’d prefer what our testing pool finds to be natural.

I suggest moving the evaluations of the non-biological datasets to the supplementary material.

**Response:** Okay – now moved!

Specific comments on the introduction:
Line 30: The Louvain algorithm is not guaranteed to produce clusters with optimum modularity because it's a heuristic that can get stuck in local minima. Suggest replacing "to produce partitions" with "a heuristic that aims to produce partitions…"

**Response:** Great point – done!

Line 31: Insert the word non-overlapping: (a collection of non-overlapping clusters)

Line 39: Rephrase. As you say below, for many datasets the answer isn't unique
Lines 49, 70, and 75: "limits on reproducibility" and later "stability" and "limits of reliability" Can you define these terms more crisply and precisely?

**Response:** We appreciate these pinpoint suggestions – very helpful and we have changed each of them

Line 777: the authors claim that for computational reasons they cap the number of labels in the network at any given time. Is this for all the networks, or only the large ones? Is this fully described in the methods section (lines 822-871)?

**Response:** Yes, this is indeed the case for all networks. We confirm this in the methods section. We’ve also added pseudo code for the whole algorithm which expresses that.

Line 879: The authors return the partition they obtain with the best NMI. Do their clusters have better NMI than alternative methods that are trying instead to optimize modularity?

**Response:** You’re correct - for many LFR networks we return higher NMI vs algorithms that try to optimize modularity. We didn’t comment on this explicitly because we aren’t sure exactly what naturalistic network community aspect of LFR networks (different than modularity) that SE2 is picking up on. It does tend to select more dense clusters (as witnessed by higher modularity density scores) but modularity density is essentially zero for these networks, so it’s probably not exactly that feature it picks up on either.

Finally, the main claim that makes Speakeasy2 so interesting, is this idea that it correctly and automatically, in a fashion similar to Louvain, will determine the "correct" number of clusters rather than require this as a separate input parameter. The authors should construct a synthetic dataset (different from the LFR benchmarks) that builds in clustering at several scales artificially. It would be super interesting to learn if Speakeasy2 always found the coarser or always found the finer partition, or if it varied by dataset, or …. I would encourage the authors to do this type of experiment to strengthen the conclusions of the paper.

**Response:** We have added the experiment you suggest – generating non-LFR network (still maintaining realistic degree distributions) with multiple simultaneous solutions on multiple scales. We also vary the density of connections across the scales, making it equal across all scales, or generating one enriched scale, which generally has the highest modularity and modularity density. As we report in an extensive section on these experiments added to the supplement there is evidence from this pool of networks that Louvain tends toward more coarse partitions, while SE2 tends toward on the partition of the enriched scale (without any parameter adjustment).

**Second round of review**

**Reviewer 1**

I appreciate the authors' efforts to address the concerns raised in the first submission. However, out of 14 points listed in the previous round of review, there are still four major issues that the authors have not addressed completely. The remaining issues are as follows:

#4. It is necessary for the authors to include a method figure that provides a high-level description of their method. The authors indicate that modified Figure 1 and the corresponding caption to address this concern, but upon review, there appears to be no change in either the figure or the caption. The authors should make sure that they submit the correct version of their paper.

#6 and #9. In the analysis results for gene expression and gene interaction networks, it would be helpful if the authors can provide some figures that are similar to the reported results on the LFR networks set to report the results. Although the authors expressed concern about the number of plots required (approximately 20), this is a research paper that requires clear, accurate, and comprehensive presentation of the method and results. There is no restriction on the number of plots that can be included in the supplementary materials. Some readers may prefer to examine the plots to better understand the obtained results, as the text in each section of the results is rather lengthy. Therefore, the authors should include plots for each test.

#7. For the single-cell experiments, it would be beneficial to present the results for each dataset and the averaged results as figures or tables (possibly in supplementary materials), in addition to text. The results are currently described in lengthy text, making it difficult to follow and evaluate. The authors can design a table to summarize the results of each separate test, the obtained value per dataset, and the averaged result. Alternatively, they could present a plot or table after each test to make the results easier to follow.

#8. The authors have revised Figure 7 as requested. However, they would like to know if the modified figure is now preferred over the original figure. As a suggestion, the authors can keep the modified figure as it clearly demonstrates the agreement between ground truth and Louvain.

**Authors Response**

**Point-by-point responses to the reviewers’ comments:**

Reviewer #1: I appreciate the authors' efforts to address the concerns raised in the first submission. However, out of 14 points listed in the previous round of review, there are still four major issues that the authors have not addressed completely. The remaining issues are as follows:

Response: Your prior comments certainly led us to improve the quality and scope of the paper, so we are happy to hear that you feel we have largely addressed the issues you raised. We also appreciate your thoughtful comments on this latest version of the paper, and believe we have substantially improved it by responding to them.

#4. It is necessary for the authors to include a method figure that provides a high-level description of their method. The authors indicate that modified Figure 1 and the corresponding caption to address this concern, but upon review, there appears to be no change in either the figure or the caption. The authors should make sure that they submit the correct version of their paper.

Response: We have multiple additions to address this request.  We've added a figure describing the typical oscillation between types of operations in the algorithm (Fig S11).  We have added a detailed schematic of every operation in the code (Figure S12) integrated with explicit calls to certain areas of the pseudocode.  For visual learners, we've also made a movie of a carefully crafted small network, and we carefully selected informative timesteps so you can see how the different stages of the algorithm play out in terms of the labels they receive, along with cluster quality.  In terms of offering a high-level overview, based on feedback from several talks, we think Figure 1 is still the best option.  We note that the very essence of the algorithm can be grasped as a simple subtraction problem, and even for that most simple operation we have a visual (Figure 1b), so that the core of the algorithm can easily be grasped by anyone in a concise manner.

Review of all types of overviews of algorithm:

S11 timeline of clustering modes - new

Figure S12 detailed flow chart of operations – new

File #1 Movie of SE2 operation on an illustrative network – new

Figure 1 (intuitive, colorful, showing essential function)

S10 pseudo code

Verbal – methods section

#6 and #9. In the analysis results for gene expression and gene interaction networks, it would be helpful if the authors can provide some figures that are similar to the reported results on the LFR networks set to report the results. Although the authors expressed concern about the number of plots required (approximately 20), this is a research paper that requires clear, accurate, and comprehensive presentation of the method and results. There is no restriction on the number of plots that can be included in the supplementary materials. Some readers may prefer to examine the plots to better understand the obtained results, as the text in each section of the results is rather lengthy. Therefore, the authors should include plots for each test.

Response: We have reduced the text length and generated many bar plots as requested for every test where it was feasible.

#7. For the single-cell experiments, it would be beneficial to present the results for each dataset and the averaged results as figures or tables (possibly in supplementary materials), in addition to text. The results are currently described in lengthy text, making it difficult to follow and evaluate. The authors can design a table to summarize the results of each separate test, the obtained value per dataset, and the averaged result. Alternatively, they could present a plot or table after each test to make the results easier to follow.

Response: We have generated many bar plots for the results and also majorly simplified the text of this section, reducing the length from 4 pages down to 2.2 pages.

#8. The authors have revised Figure 7 as requested. However, they would like to know if the modified figure is now preferred over the original figure. As a suggestion, the authors can keep the modified figure as it clearly demonstrates the agreement between ground truth and Louvain.

Response: Thank you for considering both options - we will proceed with your preferred version.

**Third round of review**

**Reviewer 1:**

The authors have made some efforts to address my concerns in their third submission. However, there is still one major issue which has not been addressed.

comment #4: There is no change made to Figure 1 and its caption. There is no new method figure has been provided. The authors indicate that Figure 1 is the best option for a high-level method figure, and it can be supported by newly added materials (Fig S11, Fig S12, File #1 Movie). However, the figure does not provide readers with a good grasp of the method. For example, in the Methods section, the authors point out that “SE2 alternates among four types of activity” but Figure 1 does not point out that there are 4 activities (modes) involved. Also, the whole process is iterative, but Figure 1 fails to capture that.

**Authors’ response**

Thank you for clarifying your request for figure 1. We added an overview of how the different components of the algorithm fit together (panel H) that also indicates they are iterative.

We appreciate your careful support in making this a clear and comprehensive study.
